# Novel Symbiotic Protoplasts Formed by Endophytic Fungi Explain Their Hidden Existence, Lifestyle Switching, and Diversity within the Plant Kingdom

**DOI:** 10.1371/journal.pone.0095266

**Published:** 2014-04-28

**Authors:** Peter R. Atsatt, Matthew D. Whiteside

**Affiliations:** 1 Department of Ecology and Evolutionary Biology, University of California Irvine, Irvine, California, United States of America; 2 Department of Biology and Institute for Species at Risk and Habitat Studies, University of British Columbia Okanagan, Kelowna, BC, Canada; Virginia Tech, United States of America

## Abstract

Diverse fungi live all or part of their life cycle inside plants as asymptomatic endophytes. While endophytic fungi are increasingly recognized as significant components of plant fitness, it is unclear how they interact with plant cells; why they occur throughout the fungal kingdom; and why they are associated with most fungal lifestyles. Here we evaluate the diversity of endophytic fungi that are able to form novel protoplasts called mycosomes. We found that mycosomes cultured from plants and phylogenetically diverse endophytic fungi have common morphological characteristics, express similar developmental patterns, and can revert back to the free-living walled state. Observed with electron microscopy, mycosome ontogeny within *Aureobasidium pullulans* may involve two organelles: double membrane-bounded promycosome organelles (PMOs) that form mycosomes, and multivesicular bodies that may form plastid-infecting vesicles. Cultured mycosomes also contain a double membrane-bounded organelle, which may be homologous to the *A. pullulans* PMO. The mycosome PMO is often expressed as a vacuole-like organelle, which alternatively may contain a lipoid body or a starch grain. Mycosome reversion to walled cells occurs within the PMO, and by budding from lipid or starch-containing mycosomes. Mycosomes discovered in chicken egg yolk provided a plant-independent source for analysis: they formed typical protoplast stages, contained fungal ITS sequences and reverted to walled cells, suggesting mycosome symbiosis with animals as well as plants. Our results suggest that diverse endophytic fungi express a novel protoplast phase that can explain their hidden existence, lifestyle switching, and diversity within the plant kingdom. Importantly, our findings outline “what, where, when and how”, opening the way for cell and organelle-specific tests using in situ DNA hybridization and fluorescent labels. We discuss developmental, ecological and evolutionary contexts that provide a robust framework for continued tests of the mycosome phase hypothesis.

## Introduction

Ancient fungi evolved an unprecedented ability to live all or part of their life cycle inside plants, joining these two lineages in an extraordinary example of coevolutionary radiation. Endophytic fungi are present in the Zygomycota (Mucoromycotina [1], [2]), Basidiomycota and Ascomycota. The majority of endophytic fungi are introduced into plants by horizontally transmitted spores, reside asymptomatically within plant tissues, and emerge during host tissue senescence. While some endophytes are easily observed within and between plant cells, the largest group (Class 3; [3]) form imperceptible infections that are apparently localized, i.e., their internal hyphal phase is limited or seemingly non-existent. These cryptic endophytes are typically discovered by DNA sequencing or by fungal isolation from small samples of cultured plant tissue [4]–[12].

Gerald Bills [13] first enumerated the many types of fungi that have endophytic forms: *plant pathogens, secondary wound invaders, epiphytic saprobes, wood decay basidiomycetes, soft rot fungi, soil saprobes, coprophilous fungi, insect pathogens and aquatic hypomycetes,* and asked, “what fungi are **not** endophytic”? This question has become increasingly prophetic as molecular probing continues to catalog the phylogenetic diversity of fungi hidden within all plants. Perhaps most perplexing, what evolutionary history would allow so many distantly related, nutritionally diverse fungi to subvert plant defense mechanisms and switch to an endophytic lifestyle? Even when internal hyphae are clearly present, there is a dearth of information about how these fungi interact with plant cells [14]. The mystery is compounded because cryptic endophytes lack a clear physical presence, yet emerge as walled cells from cultured plant tissues. The assumption that these fungi express an internal walled state is generally untested and has encouraged the default hypothesis that many endophytes persist as one or a few latent cells until they emerge and sporulate during host-tissue senescence. Yet somehow these ‘quiescent’ endophytes are biochemically coevolved [15], [16] and sufficiently active to benefit their hosts in multiple ways [3], [8], [17]–[21].

**Figure 1:**
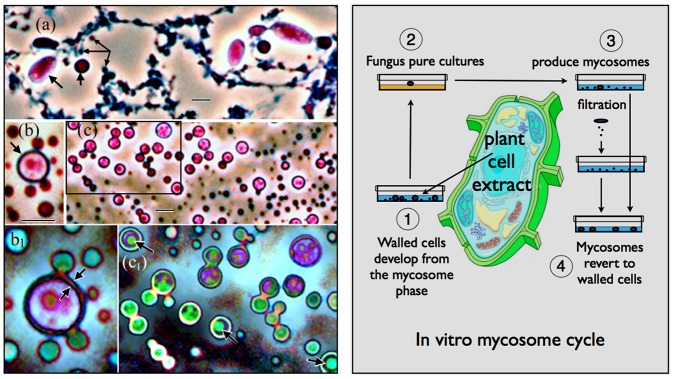
In vitro mycosome-phase culture. (a) Protoplast filaments from *Psilotum nudum* cell extract contain condensed-Ms (3 small arrows) that enlarge as spheroid protoplasts (short arrow) and *Aureobasidium pullulans* conidia (long arrow). (b and c) *A. pullulans* hyphae cultured 14 months in distilled water + erythromycin (Ms longevity test) produced condensed-Ms that divide symmetrically or by budding, and enlarge as spheroid forms that express a central vacuole. (b_1_) and (c_1_) are enlargements of (b) and the boxed area of (c); the enlargements are artificially colored with a Photoshop filter sensitive to differences in stain density. The Ms-boundary, otherwise seen as an AB-staining wall-like structure (b, arrow), is actually a narrow protoplast bounded by vacuole and plasmalemma membranes (b_1_, arrows). A walled cell potentially develops within the central vacuole-like organelle (c_1_, arrows). Bars  = 2.0 µm. **Right: In vitro mycosome cycle.** (1) Cultured in liquid media, cell extract from macerated plant tissue yields walled fungus cells that develop from the mycosome (Ms) phase. (2) Fungus pure cultures are isolated on nutrient agar. (3) Induced in liquid media, fungus cells produce Ms. (4) Ms separated from parent cells (filtered 0.8 µm) are capable of reverting to walled cells.

Here we develop a new paradigm by testing the hypothesis that endophytic fungi live within plant cells by transitioning to an endosymbiotic protoplast phase, which reverts back to the walled phase upon cell or tissue death. This concept derives from the discovery that plant cell extract contains minute chloroplast-associated bodies called mycosomes, which give rise to fungus cells [22]. When cultured in liquid media, mycosomes from *Aureobasidium pullulans* develop as filamentous and/or spheroid forms capable reverting to walled cells (Fig. 1a). Spheroid mycosomes express a central vacuole-like organelle (Fig. 1b) that forms a narrow budding protoplast (b_1_). A walled cell potentially develops within the central vacuole (c_1_ arrows). Mycosome developmental states do not stain with Cellufluor, indicating absence of cell wall beta-linked polysaccharides such as chitin or cellulose. The hypothesis that endophytic fungi can switch to an unwalled endobiotic feeding stage is supported in theory by the emerging concept [23] that Cryptomycota (diverse endoparasites with a unwalled feeding stage [24]) are related to Microsporidia and algal parasites known as aphelids. The unification of these groups establishes a new hyperdiverse clade of endoparasitic fungi near the base of the fungal tree that feed internally as unwalled protoplasts, and form a chitinous coat for reproduction and invasion [23].

Given the ambiguity of endophyte life history within plants, our goal was to evaluate whether endophytic fungi other than *A. pullulans* produce a mycosome phase. We began our study of the mycosome cycle by isolating endophytic fungi from plant anthers, fruit and stems (Fig. 1, steps 1–2). Most step 1 observations (mycosome culture from plants) will be published separately, except for an example involving *Psilotum* chloroplasts. Our experimental objective was to test pure cultures of endophytic fungi for mycosome phase formation (steps 2–3), and importantly, reversion back to the walled state (steps 3–4). A second objective was to describe mycosome phase characteristics with light microscopy. Our nascent descriptions provide a testable mycosome phase concept, and allow morphological comparisons across diverse fungal taxa. The experimental results demonstrate that diverse endophytic fungi produce a morphologically similar mycosome phase, and that some mycosome states are capable of reverting to the walled phase.

Chloroplasts apparently play a fundamental role in plant mycosome biology [22]. Identifying the functional ‘threads’ that connect chloroplasts and endophytic fungi may be key to understanding why diverse fungi live inside plants. Specifically, why would endophytic fungi, skilled at absorptive nutrition, evolve a specialized capacity to reproduce *inside* host chloroplasts? Do they acquire more than just nutrients? As deduced from electron micrographs [22], and further suggested here, the *A. pullulans* mycosome phase apparently reproduces within acquired host chloroplasts, and forms three thylakoid-associated phenotypes: electron dense bodies that can differentiate as fungus cells, single-membrane bounded protoplasts, and novel asymmetrically dividing ‘vacuolate’ units that differentiate as plastid-like organelles (Fig. 6 in [22]). Previously called “plastids that contain mycosomes (pcm)”, these plastid-like organelles develop within fungal protoplasts, and may originate from small vesicles, which are also present within the organelle envelope. Similar vesicles are present in *A. pullulans*, and considered to be a key piece of the mycosome puzzle.

*Aureobasidium* yeast cells form putative mycosomes within a double-membrane bounded organelle with internal membrane lamellae [22]; now called the promycosome organelle (PMO) to denote its central role in mycosome ontogeny. In the present work, we find that mycosomes contain a similar double membrane-bounded organelle, often vacuole-like in expression, which produces lipids or a starch grain within an inner membrane. This suggests that the PMO might represent a vacuole-like fungal symbiosome that contains a plastid vesicle. Because this organelle appears fundamental to mycosome biology, we begin the Results section with further EM illustration of the *A. pullulans* PMO, and introduce mycosome vesicles before moving to our experimental findings.

## Methods

### Experimental culture media

Because fungal mycosomes are not formed using standard growth media, we tested four non-traditional low carbohydrate liquid media (CAN, MsM, 2xT864 and Yolk-HCM) for their ability to promote mycosome formation, reproduction, and reversion to the walled state. Specific media components are given in Table S1 in File S1. Composed of citrate and ammonium nitrate, CAN was used as a non-buffered acidic medium (pH 3.6) to induce mycosome formation by fungus cells. Mycosome Medium (MsM) is a glycerol-modified fungal medium with mannitol and magnesium sulfate added as osmotic stabilizers. In some experiments, Bacto-Tryptic Soy Broth without Dextrose was prepared to manufactures specifications, autoclaved separately, and a 10 or 20 percent volume added to autoclaved MsM. After testing several PhytoTechnology plant growth media, a 2× concentration of T864 was selected. MsM and 2xT864 functioned as all-purpose media that would support mycosome formation, development and sometimes reversion. Kinetin and/or IAA (Phytotechlab.com) were added to all three media in some experiments. Yolk-HCM medium is discussed in the section describing mycosome culture from egg yolks.

### Isolation of endophytic fungi from anthers, fruit, and stems

The anthers of native parasitic plants (Indian Paint Brush, Dodder) garden plants (Natal Plum, Morning Glory, Day Lily), as well as commercially purchased fruit (Apple, Kumquat, Kiwi) were sampled for endophytic fungi (Table 1). Indian Paint Brush flower buds were sampled at Laguna Beach Moulton Meadows Park, with permission from LB Public Works Director Steve May; Dodder flower buds were sampled in the UC Natural Reserve System's San Joaquin Marsh Reserve, with permission from Faculty Manager Dr. Peter Bowler. The garden plants were sampled on the UC Irvine campus with permission of Grounds Superintendent Alfredo Mendez. Protected or endangered species were not used.

**Table 1:** Endophytic fungi isolated from plant anthers (An) fruit (Fr) and stems (St).

Tissue	Host Plant	Endophytic Fungi
An	Castilleja affinis Hook & Arn. Paint Brush.	**Filobasidium floriforme** L.S. Olive
An	Cuscuta subinclusa Dur. & Hilg. Dodder	**Cladosporium cladosporioides** (Fresen.) G.A. deVries
		Cryptococcus sp. nova
An	Carissa macrocarpa (Eckl.) A.DC. Natal Plum	**Cryptococcus victoriae** M.J. Montes, Belloch, Galiana, M.D. García, C. Andrés, S. Ferrer, Torr.-Rodr. & J. Guine
		Penicillium sp.
An	Ipomea acuminata (Vahl) Roem.& Shult. Morning Glory	**Fusarium oxysporum** Schltdl.
An	Hemerocallis flava L. Day Lily	**Trichoderma longibrachiatum** Rifai.
		**Mycosphaerella fragariae** (Tul.) Lindau
		Penicillium sp.
Fr	Malus sp. Gala Apple.	**Penicillium solitum** Westling
Fr	Fortunella crassifolia Swingle. Kumquat	**Taphrina communis** (Sadeb.) Giesenh.
Fr	Actinidia deliciosa A. Chev. Kiwi from Chile	**Cryptococcus stepposus** Golubev & J.P. Samp.
		**Cryptococcus victoriae** M.J. Montes, Belloch, Galiana, M.D. García, C. Andrés, S. Ferrer, Torr.-Rodr. & J. Guine
Fr	Actinidia deliciosa A. Chev. Kiwi from California	**Rhodotorula pinicola** F.Y. Bai, L.D. Guo & J.H. Zhao
		**Debaryomyces hansenii** (Zopf) Lodder & Kreger
		**Wickerhamomyces anomalus** (E.C. Hansen) Kurtzman, Robnett & Basehoar-Powers.
St	Psilotum nudum L. Whisk Fern	**Aureobasidium pullulans** (De Barry) G. Arnaud

All experimental fungi (bold type) produced the mycosome phase.

Plant anthers and fruit were selected because their tissues undergo natural senescence during pollen and seed development. Freshly picked tightly closed young flower buds were sequentially submerged in 70% ethanol (1 min), 0.5% NaOCl (1 min), and 70% ethanol (1 min) and allowed to dry under aseptic conditions. Using sterile forceps, two young anthers were dissected from their petal chamber, placed in 0.5 ml CAN or MsM, and macerated with a flat-tip glass rod to express pollen grains and tapetum tissues. The anther sacs were removed before adding 20 ml additional medium. Fruits purchased from commercial cold storage were surface sterilized as above (except 2 minutes in NaOCl), washed in sterile water and dried. To expose inner fruit tissue, the skin or rind was scoured with a sterile scalpel and twisted into two halves. Three tissue blocks about 5 mm^2^ were macerated in 2 ml medium and larger tissue remnants removed before adding 20 ml additional media to the cell extract. To control for fungal spore presence, 4 ml of extract-containing medium was immediately transferred to a yeast-maltose (YM) plate, allowed to settle and any remaining liquid removed. Because mycosomes do not develop on high carbohydrate ‘dry’ agar surfaces, fungus development would indicate the presence of walled spores. To document mycosome stages through time, liquid cultures were sampled immediately and at two-day intervals with bright field or phase contrast light microscopy. Efficiency was increased by covering a small pipette sample with a square cover slip, then slanting a second cover slip against the first to pull and capture excess liquid (and mycosomes) before pressing the two covers flat with paper towels. Stains were then added at cover slip margins, and pressed out again if necessary. Fungal isolates were transferred to yeast-maltose (YM) or potato dextrose agar, and experimental cell lines derived from streaked yeast cells or conidia.

### Endophytes selected as experimental fungi

Cell extract cultured from eight flowering plants and one fern (Table 1) yielded three genera of basidiomycete yeast (*Cryptococcus*, *Filobasidium*, *Rhodotorula*), and nine ascomycete genera (*Aureobasidium*, *Cladosporium*, *Fusarium*, *Mycosphaerella*, *Penicillium* and *Trichoderma*, including three yeasts, *Taphrina*, *Debaryomyces* and *Wickerhamomyces* (syn. *Pichia*). Both *Taphrina* and *Rhodotorula* are early diverging Dikarya [25]. *Rhodotorula pinicola* is described from the xylem of pine twigs [26] and wild rabbit feces [27]. *Debaryomyces hansenii* consists of a small species complex [28] closely related to *Candida guilliermondii* [29]. *Wickerhamomyces anomalus* is frequently isolated from plants, fruit, animals and soil [30]. *Trichoderma* (teleomorph *Hypocrea*) and *Mycosphaerella* (anamorph *Ramularia*) developed from mycosomes associated with *Staphylococcus warneri*, co-cultured from Day Lily anthers.

To confirm prior results in the light of new culture media, *A. pullulans* was re-isolated from the leafless fern, *Psilotum nudum* [22]. Young meristematic and older post-reproductive stems were surface sterilized (as for fruit above), washed in sterile water, cut into 1–2 cm segments, sealed moist for 7 days at 6°C (to encourage senescence and *in planta* development of the mycosome phase), and macerated in MsM. Mycosome phase development from *Psilotum* was documented, and the emerging yeast cells were transferred to YM agar for identification and experimental use. To extend our fungal sample, we briefly tested a laboratory strain of *Saccharomyces cerevisiae*, the lichen fungus *Ramalina conduplicans* [31], as well as an unidentified species of *Mucor* and *Mortierella* (Mucoromycotina). In the latter four genera we observed mycosome stages in culture, but did not attempt to demonstrate mycosome reversion to the walled state. Our endophyte sample is phylogenetically diverse (Mucoromycotina, Taphrinomycotina, Saccharomycotina, Pezizomycotina and Pucciniomycotina), and includes cosmopolitan genera (*Aureobasidium*, *Cladosporium*, *Penicillium*, *Fusarium*) with well-known endophytic members. Seven experimental genera (*Aureobasidium*, *Cladosporium*, *Mycosphaerella*, *Taphrina*, *Cryptococcus*, *Rhodotorula*, *Filobasidium*) are associated with the leaves of a single Oak species [32]. Reference to experimental fungi (Table 1) will be generic, unless more than one species is discussed.

### Mycosome formation and reversion to walled cells

Experimental fungi were induced to form mycosomes by transferring cells from YM agar (for yeast, 2 colonies approximately 5 mm each) to 100 ml liquid media (CAN, MsM or 2xT864) for 24 hr to 2 months. Mycosome development and reversion to walled cells was evaluated in three environments: (1) within cultured plant cell extract, (2) within media containing fungal pure cultures, and (3) following mycosome filtration through a 0.8 µm Gelman cellulose filter to remove parent fungal cells. Mycosomes separated by filtration were added to various liquid media and to corresponding YM agar control plates receiving 2 ml mycosome filtrate absorbed into the agar. Walled cells never developed on these control plates, confirming successful mycosome separation. Two criteria demonstrate reversion of filtered mycosomes to the walled state: formation of yeast, conidia or hyphae in association with the mycosome phase, *and* fungus reproduction when transferred to standard YM agar plates. Experimental conditions for each individual micrograph are given in Table S2 in File S1.

### Mycosome culture from chicken egg yolks

To create a lipid-rich medium for in vitro mycosome culture, fresh chicken egg yolk was added to various media. However, after discovering mycosomes within uninoculated-yolk controls, we cultured yolk-endemic mycosomes in Yolk-HCM (Yolk + High Calcium/Magnesium plant fertilizer). Each yolk was added to 300 ml sterile deionized water and homogenized, followed by transfer of a 5 ml sample to 100 ml autoclaved Technigro 15-0-15 liquid fertilizer (Sun Gro Horticulture) adjusted to 200 ppm N. In all, we sampled eighteen organic eggs from “free-range” chickens, over 10 months from three commercial sources. Each egg was brush-scrubbed with 70% ethanol, rinsed with sterile water, rotated one minute in 70% ethanol, air-dried, cracked centrally with a sterile knife blade, and the yolk passed between the two half-shells to remove the albumen. In typical experiments, the 200 ppm N solution was serially diluted 1∶1, reducing mycosome density and N levels to 100, 50, 25 and 12.5 ppm. In some experiments the dilution water contained 10^−5^ to 10^−8^ M ethylene, or 2 mg/L IAA. Mycosomes sampled for fungal DNA sequencing were grown four weeks in Yolk-HCM medium at 100 ppm N, a level at which mycosome reversion does not occur.

### Fungal identification

Two yeasts, *Debaryomyces hansenii* and *Wickerhamomyces anomalus* (syn. *Pichia anomala*) and the bacteria isolate *Staphylococcus warneri* were identified by Accugenix (Newark, DE). The remaining yeasts (Table 1) were sequenced and identified by Kyria Boundy-Mills, Curator of the Phaff Yeast Culture Collection, University of California Davis. Two *Trichoderma* ITS sequences (Genbank GU830968 and GU830969) amplified and cloned by M. Lucero (USDA Jornada Experimental Range, Las Cruces NM) showed 100% identity to *Trichoderma longibrachiatum* (EU280099). The *Mycosphaerella* isolate is 99% identical to two *M. fragariae* strains (Genbank EU167605 and GU214691) and *Ramularia grevilleana* (GU214578), the anamorph of *M. fragariae* [33]. Our isolate belongs to *Mycosphaerella* s. str., limited to taxa with *Ramularia* anamorphs [34], and is closely related to the endophytic *M. punctiformis* species complex [33]. Other filamentous fungi were morphologically identified to genus [35] or to species by ITS sequencing in our laboratory.

### DNA extraction and PCR amplification

DNA from fungal isolates and egg yolk mycosomes was extracted using a Qiagen DNeasy minikit (Qiagen, Valencia, CA) according to the manufacturer's instructions. We amplified ∼600 bp fragments of the ITS and 28S ribosomal genes with the universal fungal primers ITS1F and TW13. All fragments were amplified in 30 µl reactions using a final concentration of 1.25 µM MgCl2, 0.1 µM BSA, 0.5 µM dNTP, 0.4 µM each primer, and 0.5 U Taq DNA Polymerase (Invitrogen, Carlsbad CA). PCR was carried out with a hot start at 94°C for 3 min followed by 30 cycles at 94°C for 30 s, 52°C for 30 s, 72°C for 1 min with a final extension at 72°C for 13 min.

### Cloning and sequencing egg yolk mycosomes

The egg yolk PCR sample was diluted to a final concentration of 200 ng/ul, cloned into the 2.1-TOPO vector (Invitrogen), and transformed following manufacturer's protocols. We PCR-verified the insert size of the positive clones using universal M13 primers. All of the positive clones with inserts of the correct size and direct PCR products from mycosome cultures were sequenced (ABI 3730xl DNA sequencer, Agencourt), auto-aligned, and hand-edited to remove ambiguous bases (Geneious Pro 4.7.5). Unknown fungal clones were identified using the BLAST tool against an internal reference tree of both ITS and 28S regions separately.

### Stains and microscopy

Three stains, Melzer's reagent (MR), aniline blue in lacto-phenol (AB) and Sudan IV (SIV) allowed excellent color differentiation of mycosome states with bright field transmission light microscopy (Nikon Eclipse E600 microscope, plus Sony Digital Camera DKC5000). Images were saved at 300 dpi, and backgrounds adjusted to a neutral gray without affecting object color. AB stains fungal cytoplasmic blue; when photographed with Zeiss Axiophot phase contrast (Fig. 1), areas of AB stain appear red. MR is iodine-based, and stains fungal cytoplasm yellow, short chain polysaccharides reddish brown (dextrinoid reaction), and starch purple to deep blue. SIV is a fat-soluble dye that stains lipids, triglycerides and lipoproteins red. Fungi-Fluor (Polysciences, Inc.) contains Cellufluor, which binds nonspecifically to cell wall beta-linked polysaccharides such as chitin and cellulose. Mycosomes were stained for nucleic acids with LIVE/DEAD BacLight Bacterial Viability Kit L13152 (Molecular Probes, Inc). The kit contains two nucleic acid stains, SYTO 9 and propidium iodide (PI) that act as a Fluorescence Resonance Energy Transfer (FRET) pair. SYTO 9 is membrane-permeable, while cells with compromised membranes stain with PI. An epi-fluorescent Nikon Eclipse E400 microscope was used for phase contrast and fluorescence microscopy. Images were recorded with a Lumenera Infinity Series 1–3 digital camera and Infinity Analyze software. Electron micrographs are unpublished data from [22].

## Results and Discussion

### Mycosome ontogeny within *A. pullulans*: electron microscopy

**Figure 2:**
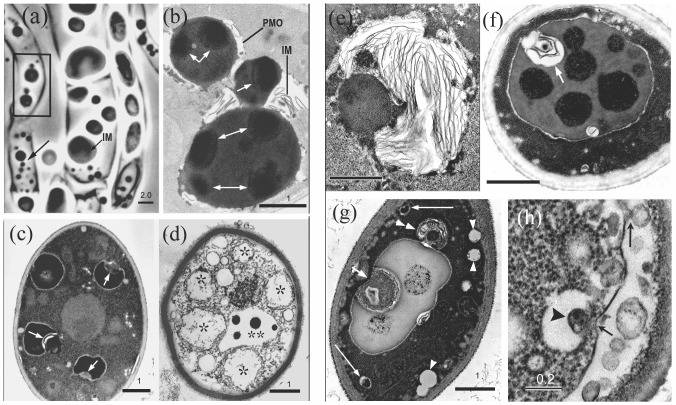
The *A. pullulans* promycosome organelle: light vs. electron microscopy. (a) Light microscopy; (b–h) electron microscopy. (a) The left hypha contains many presumptive budding PMOs (box and arrow), which express a lipoid body (arrow, center hypha) within the PMO inner membrane (IM). Walled endospores (right hypha) apparently develop within the PMO lipoid body. (b–h) First generation yeast sectioned for EM. (b) Electron-opaque budding organelles require image lightening to reveal internal bodies (white arrows) presumed to be mycosome initials. (c) PMOs are identified by invaginating membranes (arrows) and the electron-lucent space between the outer membrane and the opaque body; (see also b, f and g). (d) PMOs without lipoid content (asterisks) show numerous vesicles between the two membranes. PMOs lacking inner membrane expression may appear as vacuoles that contain electron-opaque bodies (double asterisk). (e) A mycosome-containing PMO with prominent membrane lamellae; see also (g), double arrowheads. (f) A lipoid PMO containing opaque budding mycosome initials; note invaginating membranes (arrow). (g) A lipoid PMO with a membranous infolding (short arrow) and a bud (double arrowhead) containing membrane lamellae and a putative opaque mycosome. Vacuole-like PMOs occur near the cell margin (arrowheads), some containing a putative mycosome initial (long arrows). (h) The fungal plasmalemma may bud to form periplasmic vesicles (long arrow), and may invaginate (short arrow) to form a vacuole-like PMO that contains a vesicle (arrowhead), similar to (g), long arrows. Photos (a and c) from [22] with permission. Bars  = (a) 2.0 µm; (b–g) 1.0 µm; (h) 0.2 µm.

**Figure 3:**
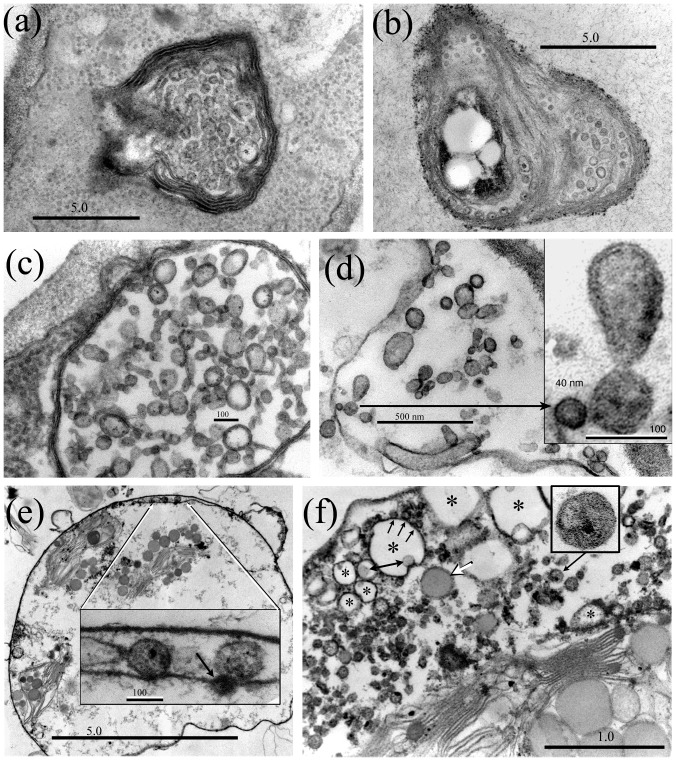
*A. pullulans* multivesicular bodies release budding Ms-vesicles. (a) Ms-vesicles are formed within multivesicular bodies (MVB). (b) Similar vesicles are present within the periplasmic space of a dividing yeast cell (tangential section). (c) Mature MVB contain large numbers of budding vesicles, which are apparently released into the periplasmic space (d). (d) Inset: An enlarged budding vesicle. (e) Similar budding vesicles are observed within the envelope of modified *Psilotum* chloroplasts that may be enclosed by a fused fungal plasmalemma (note double-thickness of the outer membrane in the enlarged inset (e), and the ‘eruptions’ from the outer membrane). An opaque vesicle associated with the plastid inner membrane (inset e, arrow) may be budding into the plastid stroma. (f) An infected plastid containing large numbers of dividing vesicles within the expanded envelope. Most of the plastid inner membrane is missing: a short segment (visible right) is lined with vesicles, two with electron lucent centers (asterisk). The vesicles enlarge as electron dense bodies (white arrow), or as vacuole-like forms (asterisks). Note budding from the ‘vacuole’ margin (3 arrows) and presence of internal electron-dense bodies (double-ended arrow). The enlarged vesicle (3f) box) was cultured from modified *Cuscuta subinclusa* plastids. Bars  = (a, b) 5.0 µm; (c) 100 nm; (d) 0.5 µm, inset 100 nm; (e) 5.0 µm, inset 100 nm; (f) 1.0 µm; boxed vesicle is 175 nm.

*Aureobasidium* yeast cells that develop from the mycosome phase continue to express stages of mycosome-forming activity. Sectioned for EM, these first generation walled cells show two distinctive organelles; double membrane-bounded promycosome organelles (PMOs) that form mycosomes (Fig. 2), and multivesicular bodies (MVB) that release Ms-vesicles (Fig. 3).

Observed with light microscopy, fungal PMOs are easily mistaken for lipid bodies, perhaps one factor contributing to their crypsis. For example, Figure 2a compares a light micrograph of presumptive *A. pullulans* PMOs to *A. pullulans* PMOs observed with EM (Figs. 2b–h). Cultured in acidic CAN medium, hyphae derived from *Psilotum* contain lipoid-bodies that apparently divide in short chains (Fig. 2a, left hypha box and arrow), while larger lipoid-bodies show contrast between a darker inner compartment (arrow, center hypha) and the outer membrane. Endospores often develop under these conditions, and are recognizable when the lipoid bodies elongate and are replaced by a developing walled cell (right hypha, see color photo, Fig. 1a in [22]). Similar electron opaque bodies observed in the EM sections (Fig. 2b) were contrast-lightened to reveal internal opaque bodies assumed to be mycosome initials (white arrows). Figure 2e illustrates a putative electron dense mycosome associated with prominent membrane lamellae, presumably formed by the inner membrane. In most sections, the two PMO membranes are separated by an electron lucent space (a vacuole formed by the outer PMO membrane), while the inner membrane encloses osmiophilic material of varying opacity (2b–c and f–g). Small vacuoles near the plasma membrane (2g arrowheads) may be incipient PMOs, two containing an opaque body (long arrows). In Figure 2h, vesicles bud from the plasmalemma (long arrow), which may also have invaginated (short arrow) as a vacuole-like organelle that contains a vesicle (arrowhead). For LM illustration of *A. pullulans* mycosome states, see Fig. S1 in File S1.

In aberrantly developing cells, the PMO inner membrane does not contain osmiophilic content (Fig. 2d, asterisks), rather sparse fibrillar material, and apparently forms numerous vesicles between the two membranes. PMOs that do not develop an inner compartment (double asterisks) may remain as vacuoles that contain opaque bodies. Vacuoles that contain opaque bodies are commonly observed in fungi [36]: during spore aging [37], during microcyclic microconidiation in heat-treated *Neurospora* [38], in dimorphic plant parasites (Fig. 5k–n, in [39]), and particularly in dimorphic opportunistic human pathogens [40], [41].

First generation yeast cells also express multivesicular bodies (Fig. 3a), which form vesicles (called Ms-vesicles) that may infect host plastids. Mature multivesicular bodies (Fig. 3c) contain numerous budding Ms-vesicles, some dividing in short chains; most apparently released into the periplasmic space (3b, d) following MVB fusion with the plasmalemma. Approximately 30–125 nm in diameter prior to release (3c), some vesicles contain a small electron opaque body, while larger forms have electron lucent centers. These budding *A. pullulans* Ms-vesicles (see enlarged 3d inset) are morphologically similar to vesicles observed within the envelope of infected *Psilotum* plastids (3e). The enlarged 3e inset shows two budding vesicles, one associated with the plastid inner membrane (arrow), presumably budding into the stroma. Note also that the outer envelope membrane is approximately twice the thickness of the inner membrane. The expanded envelope of another infected plastid (3f) contains large numbers of budding vesicles that are morphologically similar to the *A. pullulans* vesicles. A similar vesicle (3f-box inset), produced by the invaginating membranes of plastid-like organelles cultured from *Cuscuta subinclusa* cell extract, is also shown (unpublished data). Within the expanding *Psilotum* plastid envelope (3f), the vesicles apparently enlarge as two forms: electron-dense bodies (white arrow), and vacuole-like organelles (asterisk) that form small buds (3 arrows), and may also contain a dense body (double-ended arrow). Note that most of the plastid inner membrane is missing. The remaining segment (right) is lined with small vesicles, one enlarging as an electron-lucent form (asterisk).

Extracellular release of membrane vesicles by prokaryotes and eukaryotes is a conserved and underappreciated aspect of microbial life [42]. In a significant marine cyanobacterium example, *Prochlorococcus* cells release lipid vesicles containing proteins, RNA and DNA; enough to encode multiple genes [43]. Fungi also release extracellular vesicles, thus far described from several genera of opportunistic human pathogens [44]–[47] as well as *S. cerevisiae* [48]. These well-characterized vesicles are released through fungal cell walls, range in size from ca 30 to 350 nm (or 400–550 nm in SEC4 mutants) and contain a rich array of macromolecules involved in diverse processes, including amino acid/protein, sugar and lipid metabolism, cell recycling, signaling and virulence. Ms-vesicles and extracellular vesicles share many features, including size, budding, heterogeneous ultrastructure (Figs. 5 in [45], 1B in [46], 1A in [48]), perhaps lipid and nucleic acid content [49], formation within multivesicular bodies, release via budding from the plasma membrane and trans-cell wall transport [50]. While Ms-vesicles clearly require detailed analysis, their apparent presence within infected chloroplasts suggests they might function as shuttles that transport a reduced plastid cargo between chloroplasts and the walled phase; perhaps explaining our finding that a lipoid body or a starch grain is expressed within the mycosome PMO.

### Overview of mycosome concepts: light microscopy and theory

**Figure 4:**
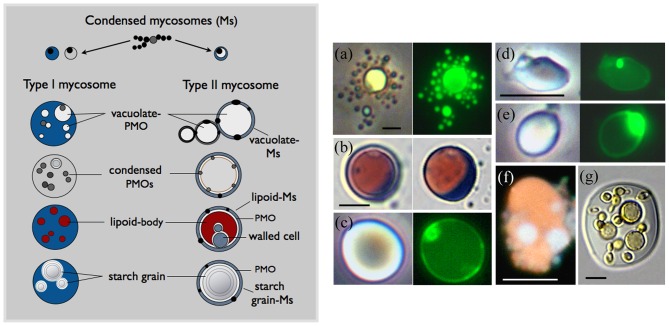
Overview of mycosome structure: light microscopy. **Left cartoon:** Condensed-Ms develop as spheroid or filamentous protoplasts that express one or more vacuole-like PMOs. Type I Ms develop as acytoplasmic (grey) or cytoplasmic (blue) protoplasts that express multiple PMOs. Type II Ms express a single PMO, bounded by a narrow budding protoplast. The PMO may appear vacuolate, or contain a lipoid compartment, a starch grain, or a walled parent-type cell. **Right: Examples of Type II lipoid-Ms.** (a) A lipoid-Ms cultured from *Cuscuta subinclusa* (yellow-pigmented) shows prolific budding (phase contrast and SYTO 9 fluorescence). (b) A *Rhodotorula* Type II Ms at two focal planes (AB/SIV stain). The red lipoid-body (left) is expressed within the non-staining vacuole-like PMO; condensed-Ms (right) are released from the crescent-shaped protoplast. (c–e) Viewed with phase contrast, refractive lipoid bodies (left) are bounded by a SYTO-9 staining envelope (right) that contains a fluorescing body. (c and e) *Saccharomyces*. (d) *Aureobasidium.* (f) An orange autofluorescing (chlorophyll-containing) lipid body from a *Psilotum* chloroplast contains two prominent DAPI-stained Ms (see also [22]; Fig. 3a–c). (g) Budding yeast-like forms within the lipoid-body of a large *Filobasidium* Type II Ms (MR stain). Bars  = 5.0 µm. Use bar d for c and e.

**Figure 5:**
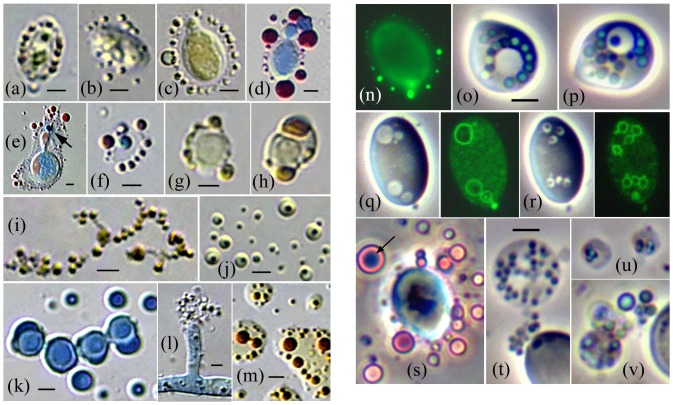
Mycosomes produced by endophytic fungi. Left panel: (a–h) Ms are associated with, or released from modified cell walls. (a–b) *Cryptococcus victoriae*, MR stain. (c–d) *C. stepposus* stained with MR (c), and AB/SIV (d). (e) An IAA-treated *Taphrina* cell enclosed by a thin expanded wall associated with lipoid-Ms and one AB-staining Ms (arrow). (f) An IAA-treated *Rhodotorula* cell showing a single AB-staining Ms. (g–i) Ms released from *Penicillium* modified cell walls (g–h) reproduced in chain-like filaments (i). (j) Condensed-Ms (here *Cladosporium*) often enlarge as spheroid protoplasts that express a dark punctate body within an internal compartment. (k) Cultured in 2xT864 medium, *Penicillium* conidia cell walls appear to expand as Ms-forming protoplasts. (l) Young *Penicillium* filaments released Ms within membrane sacs. (m) *Taphrina* Ms enlarge as protoplasts that form numerous red-brown bodies within a light-yellow staining compartment (MR stain). Bars  = 2.0 µm. **Right panel:** Phase contrast. (n) SYTO 9-stained Ms are released through *Aureobasidium* cell walls. (o–p) *Rhodotorula* cells: (o) Refractive lipoid-PMOs surround a vacuole. (p) Presumptive PMOs often contain a Ms-like body. (q–r) *Saccharomyces* cells express presumptive vacuolate PMOs with SYTO 9-staining boundaries. Note Ms release through the cell wall (q) and non-staining bodies within the PMOs (r). (s–v) *Taphrina* cells: (s) Lipoid-Ms released from a degraded cell wall (AB/SIV stain); (t) Type I protoplasts contain numerous dividing bodies, possibly PMOs; (u–v) the presumptive PMOs show refractive lipids (u), and are apparently incorporated into buds that form Type II lipoid-Ms (v). Bars in (o) and (t)  = 2.0 µm in all photos.

**Figure 6:**
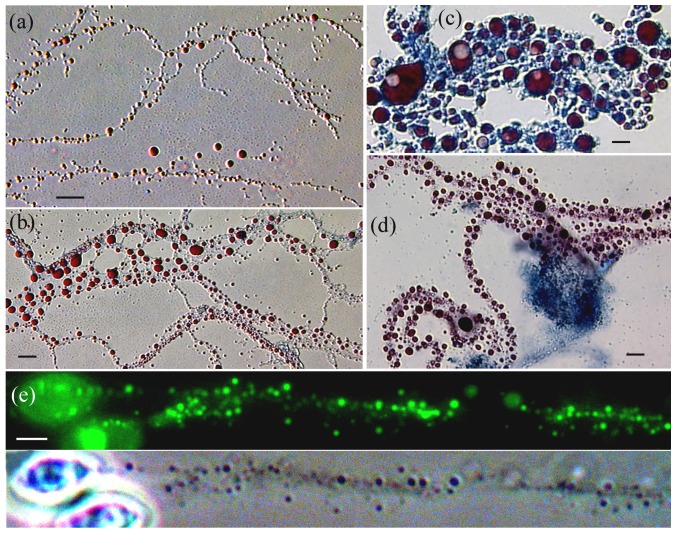
Protoplast filaments develop from filtered-Ms and conidia. (a–c) Three filament types developed from 0.8 µm-filtererd *Rhodotorula* mycosomes (AB/SIV stain): (a) Narrow reticulate filaments (<0.5 µm in diameter) produced large numbers of lipoid bodies, many pinching-off into individual spheroid-Ms. (b) Sheet-like fenestrated protoplasts may form by expansion of the PMO outer membrane. (c) *Rhodotorula* mycosomes filtered into rich media produced an AB-staining cytoplasm containing large lipoid-PMOs, some with a prominent inclusion. (d–e) Protoplast filaments from cultured *Mycosphaerella* conidia. (e) The conidia filaments presumably contain STYO 9-staining PMOs. (d) Filaments from (e) transferred to MsM-Soy over YM agar. Large numbers of SIV-staining PMOs developed within acytoplasmic (non-AB-staining) filaments, associated with an AB-staining filament mass. Bars  = 5 µm.

**Figure 7:**
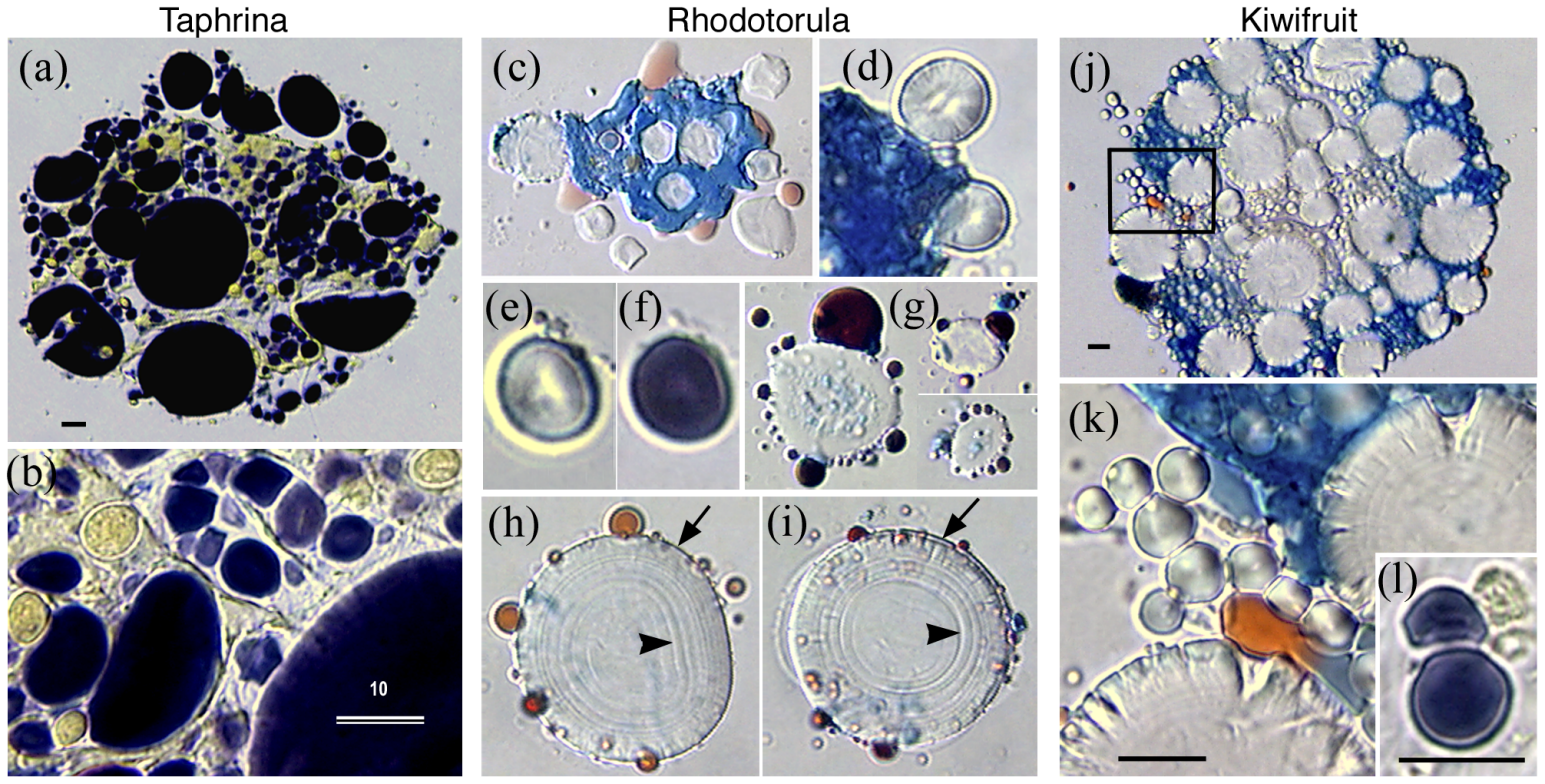
Starch grains develop within mycosomes from fungi and plants. (a–i) Starch grains from *Taphrina* and *Rhodotorula* mycosomes filtered 0.8 µm. (a–b) A *Taphrina* protoplast containing numerous starch grains and static yeast cells (MR stain). (b) An enlarged portion of (a) showing the yeast cells, some of which develop as division products of starch-Ms. (c–i) *Rhodotorula,* AB/SIV stain; (c) Cytoplasmic fungal protoplasts contain starch granules that (d) develop within the vacuole-like PMO. (e–f) A starch-Ms stained with AB (e), then with MR (f). (g) Starch-Ms often form lipoid-Ms from their boundary. (h–i) Large granules show the bounding Ms-membrane (arrows) and typical growth rings (arrowheads). (j) A fungal protoplast cultured from kiwifruit cell extract is packed with large and small Ms-starch grains (AB/SIV stain). (k) An enlargement of boxed area (j), showing chain-like division of Type II Ms-starch grains. (Inset l): A dividing Type II starch-Ms, photographed inside a kiwifruit cell (MR stain). Bars  = 10 µm. Use bar (b) for c through i.

**Figure 8:**
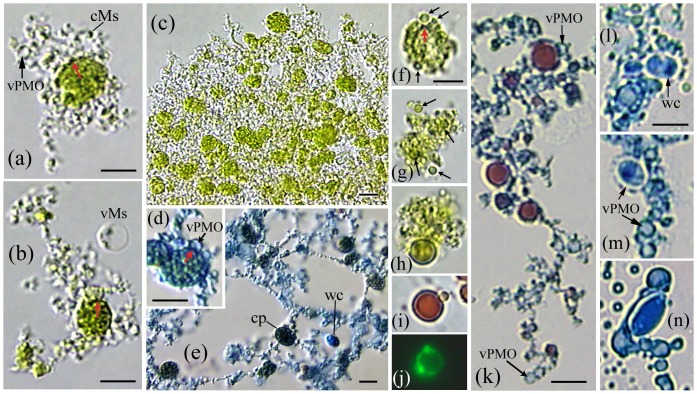
Protoplast filaments from *Psilotum* contain chloroplasts and form *A. pullulans* cells within PMOs. (a–b) Protoplast filaments develop from the margin of *Psilotum* chloroplasts; note that portions of the plastid envelope contain dark punctate bodies (also f, red arrows). (a) Vacuolate-PMOs (vPMO) enlarge within the filaments, and (b) pinch-off as spheroid vacuolate-Ms (vMs). (b) Filaments radiating from chloroplasts also contain small chloroplasts. (c) The filaments mature as a dense chloroplast-containing matrix; or (e), expand as fenestrated, chloroplast (cp)-containing filaments (AB stain). Walled cells (wc) occasionally develop within the ‘open’ areas. (d) vPMOs apparently develop from the AB-staining chloroplast margin (red arrow). (f–h) *Psilotum* chloroplasts release green Ms (black arrows) from outside a dark chloroplast envelope (f, red arrow), and from green unbounded membranes (g–h). (h) This Type II Ms contains a greenish body, similar to *Psilotum* lipoid-Ms that stain red (i) with SIV. (j) SYTO-9 staining bodies are present within the Type II protoplast. (k–n) Filaments from post-reproductive stems contain numerous vPMOs (k), some expressing a lipoid body (AB/SIV stain). (l–n) *Aureobasidium* walled cells (wc) develop within the PMO vacuole. (n) Note presence of condensed-Ms, enlarging vacuolate-PMOs and yeast cell release from a vacuolate-PMO. Bars  = 5.0 µm.

**Figure 9:**
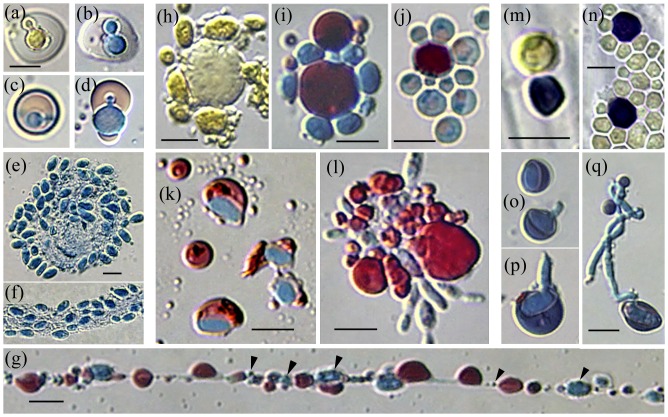
Mycosome reversion to walled cells. (a–d) Type II lipoid-Ms often contain budding yeast-like bodies that do not revert to parent cells. (a) *Taphrina*, MR stain. (b) *Penicillium*, AB stain. (c) *Fusarium*, AB/SIV stain. (d) *Rhodotorula*, AB/SIV stain. (e–f) Ms filtered from IAA-treated *Rhodotorula* formed walled yeast cells within spheroid (e) and filamentous (f) protoplasts. (g) IAA-treated *Taphrina* cells produced narrow filaments that formed lipoid-Ms and yeast cells, AB/SIV stain. (h–j) Walled cells develop from the margin of Type II lipoid-Ms: (h) *Filobasidium*, MR stain; (i) *Rhodotorula* and (j) *Wickerhamomyces*, AB/SIV stain. (k) *Trichoderma* conidia developed within lipoid-Ms, AB/SIV stain. (l) *Taphrina* yeast cells originating within a cluster of lipoid-Ms, AB/SIV stain. (m–n) Walled cells develop as division products of starch-producing Ms, MR stain. (m) A *Penicillium* conidium observed within an apple cell, MR stain. (n) Multipolar budding of *Wickerhamomyces* yeast from starch-Ms within a kiwifruit cell. (o–q) *Cladosporium* conidia germinate within mycosome PMOs. Bars  = 5.0 µm.

**Figure 10:**
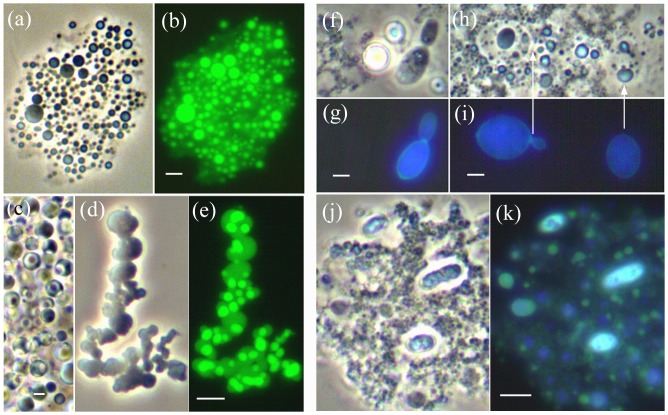
Mycosomes from egg yolk revert to walled cells. (a–b) Yolk-Ms show prolific reproduction within thin membrane sacs. (a) Phase contrast, (b) SYTO 9 stain. (c) IAA-treated Ms formed large membrane-sacs containing numerous spheroid protoplasts, each with a prominent central body. (d–e) Ms cultured in MsM flooded over Sabouraud dextrose agar. The variable-shaped lipoid bodies (d) contain (e) fluorescing PMOs or Ms (SYTO 9 stain). (f–k) Cell wall formation by *Rhodotorula glutinis* stained for chitin with Fungi-Fluor. (f and h) Yeast cells were present at 38 days within a diffuse protoplast matrix containing lipoid-Ms (phase contrast). (g and i) Ms and the protoplast matrix do not express chitin; only the mature cell (g) and thin walled yeast cells (i) fluoresce. (j–k) Sampled two weeks later, three walled cells and many enlarging Ms show variable cell wall fluorescence. Bars  = 2.0 µm.

The Figure 4 cartoon illustrates mycosome developmental states inferred from light microscopy (LM). Condensed-mycosomes (cMs) released from parent fungal cells are hypothesized to be plasma membrane-bounded acytoplasmic protoplasts that contain a double membrane-bounded PMO, considered homologous to the *A. pullulans* PMO. Our LM descriptions are based on this three-membrane model. Because the single membrane difference between a condensed-Ms and its PMO cannot be detected with LM, some bodies released from fungus cells could be vesicles or organelles rather than protoplasts. However, we can rule out subcellular units when the condensed-bodies show expected patterns of mycosome differentiation and reach ∼10–20 µm in diameter (Figs. 4–10), some double that size (Fig. S4 in File S1).

Condensed-Ms differentiate as two types, distinguished by number of PMOs: Type I Ms may be spheroid, amorphous or filamentous, and typically express many PMOs. Type II Ms contain a single large PMO, typically surrounded by a narrow AB-staining cytoplasm. Thus mycosomes have a filamentous phase and a spheroid yeast-like phase (Type II). The PMO may appear vacuolate, or contain a lipoid compartment, a starch grain, or a walled parent-type cell. Type II-Ms develop directly from condensed-Ms, or from Type I spheroid or filamentous protoplasts that bud or pinch-off an organelle-containing protoplast. Type II Ms are referred to as vacuolate-Ms, lipoid-Ms, or starch grain-Ms.

Condensed-Ms (∼0.5–1.0 µm) are difficult to comprehend as fungal cell precursors, particularly because the mycosome phase is multifunctional, and we do not know specifically how or when mycosomes acquire parent nuclei or mitochondria. A basic working model predicts that Ms-vesicles, PMOs, and the plastid-like organelles from *Psilotum* chloroplasts may be different functional states of the same organelle. Because each unit apparently reproduces by budding, we suspect that Ms-vesicles contain a small genome independent of the parent nucleus required for reversion to the walled state. Some fraction of PMOs should also contain parent nuclei, since walled cells develop within this organelle.

Lipid expression within the mycosome PMO is exceedingly common; some examples are given in Figure 4, right panel. Prolific budding (a) is a characteristic mycosome trait, often accompanied by nucleic acid (a) or DAPI-staining (f). Mycosome states cultured from plant cell extract often contain pigmented lipids that match the color of host plastid pigments: (a) yellow *Cuscuta* carotenoids, or (f), orange autofluorescing chlorophyll within *Psilotum* plastoglobule-like bodies. Type II lipoid-Ms are clearly identified when a red (SIV-staining) lipoid body is present within the non-staining vacuole-like PMO (b, left), enclosed by a narrow or crescent-shaped budding protoplast (b, right). Some refractive lipoid bodies cultured from fungi did not show Type II morphology. These units are enclosed by a SYTO-9 staining boundary (c–e), and may represent PMOs released into the medium. Some are apparently nucleated (c–e); however most did not contain a fluorescing body. The large *Filobasidium* Type II-Ms (g) may show mycosome transition to budding yeast-like cells within the lipoid PMO. The full breadth of mycosome phenotypic variation includes giant protoplasts that develop in clusters, and apparently form a walled compartment within each protoplast envelope (Fig. S4 in File S1). As judged by DAPI staining in the Figure S4j culture, less than half of the condensed-Ms were nucleated.

### Mycosome phase from endophytic fungi

All experimental endophytic fungi (Table 1) produced the mycosome phase in one or more liquid media, particularly MsM and 2xT864. Condensed-Ms may exit through fungal cell walls (Fig. 5a–d), as do extracellular transport vesicles [48]. However, in most treatments the fungal cell wall was highly thinned (5b, e) or degraded (5s); or release occurred in membrane sacs, presumably through cell wall openings (5l, and see Fig. S1f in File S1). Cultured in CAN, mycosomes (or PMOs) from *Penicillium* conidia apparently remained within modified cell walls (5g–h); whereas in 2xT864 medium the *Penicillium* wall appears to expand into AB-staining segments that seemingly pinch off as mycosomes (5k). The condensed-bodies released from fungus cells show characteristic mycosome developmental states. They reproduce in chain-like filaments (5i), wherein PMOs presumably divide within a close-sheathing plasmalemma. Alternatively, they enlarge as plasma membrane-bounded spheroid protoplasts (5j) that show a dark punctate body within an internal membrane, presumably the PMO. Within enlarging *Taphrina* protoplasts (5m), PMOs apparently bud to form numerous reddish-brown staining organelles (dextrinoid reaction to iodine-containing MR stain). Significantly, as realized during data analysis, the single AB-staining mycosome formed by *Taphrina* cells (5e, arrow) occurred in MsM containing 2 mg/L IAA. Similarly, cultured in CAN +1 mg/L IAA, many acytoplasmic *Rhodotorula* cells contained a single AB-staining mycosome (5f). AB-staining condensed mycosomes were not otherwise observed in direct association with parent cells.

Cultured in MsM +20% Soy, *A. pullulans* yeast cells (Fig. 5n) released SYTO 9 staining mycosomes (for detailed response to another medium, see Fig. S1 in File S1). Vacuoles hypothesized to represent the PMO outer membrane were often observed within parent cells (5o–r), some containing a punctate body (5p, r). In *Rhodotorula*, lipoid-PMOs surround a parent vacuole (5o); in *Saccharomyces* grown from glycerol-frozen stock in CAN-MsM-Soy, budding vacuolate-organelles show a SYTO 9-staining boundary and apparently release condensed-Ms through the cell wall (5q–r). Cultured in MsM, *Taphrina* cells released typical Type II Ms that contain an AB-staining body within the SIV-staining lipoid compartment (5s, arrow). In MsM without glycerol, *Taphrina* cells produced Type I Ms that apparently contain many dividing PMOs (5t). The presumptive PMOs develop refractive lipids (5u) and may be incorporated into buds that form Type II lipoid-Ms (5v).

### Lipoid bodies within protoplast filaments

Figure 6 illustrates the prodigious number of SIV-staining lipoid bodies expressed within Ms-filaments, presumably within PMOs. *Rhodotorula* parent cells cultured in CAN + IAA formed mycosomes (separated by 0.8 µm filtration) that developed three filament morphologies in different media (see Table S2 in File S1). Figure 6a shows a small sample of narrow filaments (∼0.5 µm in diameter) that expressed hundreds of lipoid PMOs, many of which pinch off and may develop as Type II spheroid-Ms (recall Fig. 4b). In a second medium, the mycosomes developed as sheet-like fenestrated protoplasts with variable sized lipoid-bodies (6b). Filtered into rich media, the *Rhodotorula* mycosomes formed cytoplasmic (AB-staining) filaments that expressed large PMOs with an AB-staining boundary, some with a vacuole-like inclusion (6c). *Mycosphaerella* conidia cultured in MsM +20% Soy (Fig. 6e), produced thin membranous filaments containing numerous SYTO 9-staining PMOs. When transferred to fresh media over YM agar for 24 hr, these acytoplasmic filaments produced prolific numbers of lipoid-PMOs (6d), associated with an AB-staining filament mass. Direct protoplast filament formation by *Mycosphaerella* conidia suggests one pathway for mycosome phase colonization of plant cells.

### Starch grains in mycosomes from fungi and plants

In the 2003 study, starch grains were consistently associated with yeast cells cultured from *Psilotum* cell extract (Figs. 3–4 in [22]), and were presumed to originate from host plastids incorporated into fungal protoplasts. However, a more complex ‘plastid-within-mycosome’ hypothesis (Fig. 4e–j in [22]) was supported here when semi-crystalline starch grains surprisingly developed *inside* mycosomes filtered 0.8 µm from pure cultures of endophytic fungi. Indeed, starch grains sporadically developed within the PMO of mycosomes filtered 0.8 µm from all routinely cultured experimental fungi (*Rhodotorula, Cryptococcus, Filobasidium, Taphrina, Cladosporium, Mycosphaerella and Aureobasidium*), including two cultures of egg yolk mycosomes.

Figure 7 illustrates Ms-starch grains from *Taphrina* and *Rhodotorula*, and for comparison, from kiwifruit cell extract. Starch grains were often present in fungal protoplasts (Fig. 7a), which also contained static, non-budding yeast cells (7b). *Rhodotorula* cells cultured in 2xT864 +IAA produced starch-Ms within and protruding from the margins of AB-staining protoplasts (7c–d). At median focus, the narrow protoplast and PMO vacuole are apparent (7d). PMO morphology is also illustrated by a single mycosome photographed after protoplast staining with AB (7e), and again after staining for starch (7f). Individual grains were numerous, and produced SIV-staining lipoid-Ms from unapparent bounding membranes (7g). Larger grains (7h–i) show lipoid-Ms associated with a discernible Ms-boundary (arrows), as well as the prominent growth rings (arrowheads) that characterize plant starch [51]. Large AB-staining protoplasts observed in cultured kiwifruit extract (7j) are similar to starch-containing protoplasts cultured from fungus-derived mycosomes (7a). The former contain large grains as well as smaller Type II starch-Ms, many dividing in chain-like fashion (7j-box enlarged in (k). Ms-starch observed within kiwifruit cells (7l inset) and other plants can be distinguished from plant starch by Type II morphology (grain presence within the PMO), division pattern, and division products that express lipids, starch, or a fungus cell.

### Mycosome phase from plants: the *Psilotum* example

Mycosome developmental states were observed in all cultured plant extracts that produced endophytic fungi (Table 1). With appropriate media and tissue sampling, several stages of the *Psilotum* mycosome phase can be observed in vitro (Fig. 8). Cultured in MsM, extract from young meristematic stem tips (without cold treatment) produced fungal filaments that contained host chloroplasts (Fig. 8a–e). Viewed at high magnification, portions of each chloroplast envelope apparently contain dark punctate bodies (8a, b, f, red arrows), which could represent Ms-vesicles (recall Fig. 3e–f). If Ms-vesicles enlarge as vacuolate-PMOs (vPMOs), this may explain vPMO development at the chloroplast margin (8d), and within protoplast filaments (8a, k).

Young pre-reproductive stems sampled below the meristematic tips (cold-treated and cultured in MsM +20% Soy), produced remarkable images of individual chloroplasts releasing greenish mycosomes (arrows) from a layer outside the dark punctate plastid envelope (Fig. 8f, red arrow). Similar mycosome stages were associated with unbounded, tightly clustered green (thylakoid-like) membranes (8g, h). The larger lipoid-Ms (8h) may contain chlorophyll (see Fig. 4 in [22]) and is morphologically similar to Type II SIV-staining lipoid-Ms commonly isolated from both *Psilotum* (8i, j) and endophytic fungi (Fig. 4b). Filaments cultured from older post-reproductive (cold treated) stems contained numerous vacuolate PMOs (8k–n): some expressing an SIV-staining lipoid-body (8k), others forming an *A. pullulans* yeast cell within the vacuolate PMO (8l–n). In sum, these in vitro developmental states are consistent with the hypothesis that mycosomes reproduce within host plastids acquired by the *A. pullulans* protoplast phase [22]. Moreover, the cultured protoplast filaments express a lipoid body within the PMO, the same vacuole-like organelle within which walled cells develop. This pattern (minus chloroplasts) is fundamentally similar to in vitro mycosome culture from endophytic fungi, as detailed below.

### Mycosome reversion to walled cells

Mycosomes from both plants and fungi reverted to walled cells (Fig. 9). However, plant mycosomes reverted most consistently, presumably because this transition naturally occurs during plant senescence, and required stimuli were present. In addition to *Psilotum* stem extract, mycosome transition to walled cells was easily observed in senescing fruit, particularly within cultured apple cells (*Penicillium*, Fig. 9m), and kiwifruit cells (*Wickerhamomyces*, Fig. 9n, and *Cryptococcus*, not shown). Anther tapetum cultures also produced walled cells, both yeast and filamentous forms (Table 1). Day Lily anther tapetum cultures contained mycosome protoplasts that co-cultured with *Staphylococcus warneri*. This bacteria-mycosome consortium gave rise to both *Mycosphaerella* and *Trichoderma*, the latter originating from AB-staining conidia that developed within red SIV-staining mycosomes (Fig. 9k; the *Staphylococcus* cells are not present in this particular photo). In general, Figure 9 illustrates two pathways of mycosome reversion to the walled state; endogenous development within the PMO, and budding from the margin of lipoid- or starch grain-Ms.

In contrast to plants, mycosomes produced by fungi and separated by 0.8 µm filtration usually failed to revert to viable walled cells. Lipoid-Ms were often observed, and while some contained budding yeast-like forms (Fig. 9a–d), these developmental states were not culturable when transferred to YM agar. In two experiments, fungal mycosomes filtered into *Psilotum* chloroplast-rich cell extract reverted to walled cells (e.g., *Filobasidium* Fig. 9h, and *Taphrina*, Fig. 9l), whereas *Psilotum* control extracts without mycosome filtrate were negative for fungi. This suggests that plant extract contains chemical stimuli that may trigger reversion of nucleated mycosomes.

In later experiments, yeast cells added to 2xT864 medium plus 1–2 mg/l IAA formed mycosomes that reverted to walled cells in the conditioning medium. While it was difficult to assess mycosome-to-yeast transitions in the presence of mycosome-forming parents, directionality was clear when the walled cells developed within narrow protoplast filaments (*Taphrina*, Fig. 9g), or as multiple buds from lipoid-Ms (*Rhodotorula*, 9i). Filtered 0.8 µm into bovine serum albumin, mycosomes from *Rhodotorula* cells treated in CAN + IAA produced large numbers of yeast within ovoid protoplasts and long strand-like filaments (Fig. 9e–f). One month later, a second 0.8 µm filtrate from the same parent cells, flooded over YM agar, formed yeast that apparently developed directly from individual mycosomes, and many yeast divided to form walled filaments. While paired controls without IAA were not included in these experiments, the very large set of previous failures in similar media strongly suggest an IAA reversion effect (see also Fig. 5e, f). In retrospect, testing the specific role of IAA in mycosome formation and reversion should be rewarding.

### Mycosomes from egg yolk revert to walled cells

Mycosome discovery within chicken egg yolk provided an independent system for validating the mycosome concept. Egg yolk mycosomes cultured in high N (100–200 ppm, observed for 2–3 months) never reverted to walled cells, and reverted only sporadically in low N treatments (25 or 12.5 ppm N). Ten cloned samples of PCR amplified DNA from a high N, non-reverting mycosome culture identified three fungi: *Aspergillus versicolor* sequences were recovered from 7 clones, *Fusarium udum* from 2 clones and *F. nygamai* from one. Fungi that reverted in low N cultures included *Chaetomium globosum*, known as a pathogen, saprotroph and endophyte, *Cladosporium cladosporioides*, *Penicillium toxicarium*, *Rhodotorula glutinis* (or *Rhodosporidium* [52], and a probable species of *Teratosphaeria* with 96% similarity to *T. knoxdavesii*. Non-sequenced isolates included two yeasts, one white, one hyaline glossy, and 8 filamentous fungi morphologically identified to genus: *Penicillium* (4), *Cladosporium* (2), *Aspergillus* (1) and *Fusarium*.

All yolk samples (n = 18 eggs) contained abundant mycosomes. At 50–100 ppm N, the mycosomes multiplied within thin membrane sacs (Fig.10a–b), producing an easily visible, cloudy white population sustainable for several months. This typical budding morphology can be altered by media differences: in the presence of 2 mg/L IAA the membrane sacs were very large and contained numerous spheroid protoplasts, each with a prominent central body (10c); in MsM flooded over Sabouraud dextrose agar, variably-shaped lipoid-bodies contained unapparent mycosomes visible with SYTO 9 staining (10d–e). These reproductive states did not lead to the formation of walled cells. Stages of mycosome reversion were observed when a thin protoplast matrix developed, containing lipoid-Ms (10f, h). Stained for chitin with Fungi-Fluor, neither the membrane matrix nor the lipoid-Ms fluoresced (10g–i), while mycosome-derived *Rhodotorula glutinis* yeast showed cell wall presence, even in early transparent stages (10i, arrows). Twelve days later, variable chitin fluorescence was associated with numerous small mycosomes ranging up to thick-walled yeast cells (10j–k).

### Implications of a cryptic endosymbiotic phase within fungal life cycles

Current concepts of plant-fungal interactions derive from the view that fungi are “outsiders” that continually besiege the plant fortress, breech defenses and adapt in ways that decrease (parasitism), increase (mutualism) or have no observed influence (commensalism) on plant fitness. While pathogen principles are universal and well tested, this perspective may overshadow other possibilities; e.g., if the mycosome phase is ancient, broadly conserved, and present in all plants, fungi may have been “insiders” from the beginning, cycling between a broadly coevolved protoplast phase and an independent walled state. We infer that individual fungal genomes may be simultaneously adapted to remarkably different lifestyles, e.g., free-living parasite, saprobe, coprophile etc. on the one hand, and unwalled endosymbiont on the other. The protoplast and walled phase are presumably governed by different sets of regulatory genes, similar to phase-specific genes expressed in the yeast-hyphal dimorphism, in anamorphs and teleomorphs, or in plant sporophytes and gametophytes [53]. Adaptations expressed by one phase would not limit the other.

### Phase cycling and opportunistic fungal adaptation

The phylogenetic and ecological diversity of endophytic fungi can reasonably be explained by the ancient origin of a heritable mycosome phase, plus opportunistic adaptation that may derive from interplay of the two life history states. Plants provide ubiquitous and ever-changing niches for new mycosome phase adaptations, and also provide ‘sustained proximity’ [54] to extreme environments, both biotic and abiotic [55], creating fungal reservoirs for continuous stepwise selection of genetic variants expressed by the walled phase. Thus the independent walled phase may diverge rapidly (i.e., switch lifestyles often, with or without morphological change), while the supporting protoplast phase may evolve more conservatively in response to endosymbiotic selection pressures.

This dual level of opportunistic selection predicts that most endophytes should retain both phases; e.g., endophytes with a well-developed internal walled state may retain an abbreviated mycosome phase or vestiges thereof, and most ‘non-culturable’ fungal endophytes may in fact have a walled state. Generic relatives of known endophytes may also retain this phase. The genus *Trichoderma*, with only a few described endophytic species, has a remarkable range of lifestyles and interactions with other fungi, animals and plants [56], including molecular cross talk that underlies dramatic plant benefits [57]. Testing a diverse sample of these opportunistic species for mycosome formation (including mycoparasites) would be illuminating. The mycosome phase (or some vestige) may be indispensible for plant symbiosis.

Since mycosomes have long remained asymptomatic and undetected within plants, the same may be true for animals that ingest plants or plant detritus, as suggested by mycosome presence within chicken egg yolks. Egg yolk mycosomes reverted to common endophytic genera, many prevalent in poultry feed [58], [59] and in the airborne micro flora of poultry houses [60], [61]. Ingested hyphae or spores may produce the mycosome phase during digestion, or mycosomes may be ingested directly from plants or poultry feed (cereal endophytes) and then follow a path similar to *Salmonella enteritidis* into the egg yolk via the reproductive tract [62]. If plants function as ‘grand reservoirs’ for mycosome phase ingestion and dissemination by animals, rapid nutritional shifts, opportunistic adaptation, and interkingdom host jumping might be expected outcomes [54], [63]–[65]. This cryptic transmission syndrome (probable mycosome phase presence in water, pollen, plants and animals) may explain the prevalence of opportunistic human pathogens, which occur in 11 mycosome-forming genera (*Cryptococcus*, *Rhodotorula*, *Fusarium, Penicillium, Trichoderma*, *Cladosporium, Wickerhamomyces* [*Pichia*], *Debaryomyces*, *Chaetomium, Aspergillus and Aureobasidium*). Of significant medical importance, this subject will be discussed separately.

### Host chloroplasts: key to the mycosome puzzle

While a plastid-like organelle within the PMO remains to be demonstrated, the congruence of lipids and water-insoluble granular starch within this organelle is difficult to explain without assuming some form of plastid presence. Fungi produce amylose, but not amylopectin, the semi-crystalline branched glucan polymer that provides about 75% of the granule mass. In green algae and plants, crystalline amylopectin synthesis occurs in plastids and requires the concerted action of ADP-glucose pyrophosphorylases, starch synthases, branching enzymes and starch-debranching enzymes [66], [67]. Like an elephant in the room, starch grain presence within the fungal PMO demands explanation. Discovery of a new chloroplast-derived organelle, the “tannosome” [68], provides a functional model for exploration. Tannin-producing vesicles derive from thylakoid membranes that pearl into 30 nm spheres, which are then encapsulated into chloroplast shuttles formed by fusion of both envelope membranes. The plastid shuttles move into invaginating plant vacuoles, forming a double membrane-bounded organelle (Figs. 5, 7 in [68]). It seems unlikely that tannin production could be the sole selection pressure for thylakoid segmentation into vesicles that are transported into vacuoles by plastid shuttles. This elaborate plastid deconstruction mechanism might also form minimal plastid units that are transferred into Ms-vesicles, which in turn may differentiate as plastid-like organelles (Fig. 6 in [22]). Now enclosed by a fungal Ms-vesicle membrane, these “mycoplasts” (fungus-plastids) may have immediately increased plant fitness by functioning in specialized cells, tissues or starch producing storage organs (e.g., fruits, storage roots, tubers, rhizomes, seed cotyledons etc. [69]). Maintained by the plant genome, biochemically primed mycoplasts might also function as double membrane-bounded PMOs during mycosome reproduction, and would thus be transferred into the walled phase during reversion. In theory, this plastid-centric mutualism could be the basis of an enduring plant-fungal partnership. Clearly, an in-depth understanding of mycosome life history within plants is crucial to interpreting their biology and evolution.

### A zoosporic context for mycosome evolution

How did mycosomes evolve? Notably, the zoosporic antecedents of terrestrial fungi [70], [71] express most traits necessary to explain mycosome evolution. In short, mycosomes and zoospores lack a cell wall and require water, are similar sized propagules (small zoospores are ca 0.5–1.0 µm [72]) formed within a sporangium-like sac, and given appropriate chemical signals, switch from protoplasts to walled cells. Both also depend upon lipid reserves, in zoospores often a single large lipid body, perhaps evolutionarily replaced by a plastid-derived lipoid body within the PMO.

Which zoosporic lineage might have led to an unwalled phase within terrestrial fungal life cycles? Early endoparasitic fungi (Cryptomycota) will have much to tell, particularly lineages such as *Amoeboaphelidium* [73] and *Rosella* [70], [74], where walled parasites shift to an endobiotic protoplast phase that develops in direct contact with the host cytoplasm; and in *Rosella*, may incorporate host cytoplasm and organelles via phagocytosis. While the genetic roots of the mycosome-phase are presumably ancient, we focus here on *Olpidium*, a relatively recent clade of zoosporic plant parasites whose plasma membrane is also in direct contact with the host cytoplasm, presumably increasing plant ability to regulate the parasite [70].

Surprisingly placed within the polyphyletic Zygomycetes in 2006 [25], [68] and recently confirmed by Sekimoto et al. [75], *Olpidium* is now seen as the closest living flagellated relative of terrestrial fungi, leaving open the possibility that it may be sister to Dikarya, or to one of the basal clades within Zygomycota. Significantly, *Olpidium* is an *asymptomatic* nonpathogenic “parasite” [76]; in effect, a flagellated endophyte of terrestrial plants. In their description of *Olpidium* sporogenesis, Temmink and Campbell [76] recognized three morphologically distinct units: vacuole, multivesicular body and osmiophilic body, which they considered to be variations of a single pleomorphic organelle, referred to as vacuoles when empty, or as multivesicular bodies if they contained small vesicles and/or osmiophilic deposits, presumably lipid. The *Olpidium* multivesicular bodies both accumulate and release lipid material, and prior to sporangial cleavage these “empty” organelles widen and fuse to form the cleavage vacuoles that separate zoospore protoplasts. Notably, this organelle occurs throughout the vegetative growth of *Olpidium*, obscured by electron dense deposits that hide the internal vesicles or the vacuole membrane. Does *Olpidium* contain a predecessor to the mycosome PMO? Perhaps a member of the *Olpidium* clade underwent host-regulated developmental changes (delayed sporangium wall development, intracellular zoospore release, and flagella loss) to yield the mycosome prototype: an asymptomatic protoplast phase within plant cells, preadapted to cycle back to walled cells capable of regenerating the endophytic phase.

### Testing mycosome antiquity

Irrespective of how mycosomes evolved, if this cryptic life history state is ancient, then most fungi symbiotic with phototrophs (algal endophytes, lichen fungi, plant endophytes and endo-ecto-mycorrhizal forms) may express this phase or retain vestige states. Quite possibly, the mycosome phase may have evolved within green algae, or earlier within cyanobacteria that gave rise to chloroplasts. If lichen-forming fungi evolved from endophytic ancestors, their algal symbionts may vertically transmit the mycosome phase of the lichen mycobiont. Three observations support this hypothesis: mycosome formation by the lichen mycobiont *Ramalina conduplicans* (Figure S2 in File S1); the isolation of a *Cladonia cristatella*-like mycobiont from a curated lineage of the lichen alga *Trebouxia erici*; and culture of putative mycosome states from cyanobacteria and green algae [22]. Armed with new questions and predictions, fungal presence indicated by DNA sequencing can now be followed by cell or organelle-specific localization using in situ DNA hybridization or fluorescent labels.

Testing both Mucoromycotina and Glomeromycota for this phase may begin to unravel mycosome evolution in terrestrial plants, and perhaps broaden the developing Mucoromycotina perspective [1], [2] regarding the earliest fungal architects of the land plant symbiosis. In addition to Glomeromycota symbionts, basal liverworts and hornworts contain diverse Mucoromycotina, including *Endogone*-like endophytes with intracellular coils and thin-walled ‘swellings’. In both liverworts and hornworts, published electron micrographs contain unidentified structures. For example, small electron opaque vacuolate forms, some associated with lipid bodies (Fig. 3a arrows, in [77]), and electron dense bodies (Fig. 1e–f in [2]), might be considered as possible candidates for mycosome states. Mucoromycotina endophytes are increasingly reported in vascular plant roots (e.g., *Absidia*, *Dissophora*, *Umbellopsis* and *Zygorhynchum*), including *Mucor* and *Mortierella*, which await testing for mycosome reversion capability (Fig. S3 in File S1).

Vesicular-arbuscular mycorrhizal fungi presumably evolved from endophytic forms [78], [79]. Indeed, Glomeromycotan fungi often grow as endophytes (i.e., without a localized interface of specialized hyphae) and produce lipoid vesicles only [80], [81]. Lipoid vesicles formed by symbiotic fungi may represent evolutionary vestiges of mycosome ancestry. Dark septate root endophytes contain SIV-staining ‘vacuoles’ that we assume to be lipoid-PMOs, as well as large terminal vesicles (Fig. 7 in [82]) that may contain Type II lipoid-mycosomes, one showing a division plane. Best known as a soil saprobe, *Endogone pisiformis* produces lipid-filled hyphal vesicles when growing in and on plant surfaces [83], and appears to release mycosome-like bodies (Fig. S5 in File S1). The unusual ‘swellings’ formed by *Endogone*-like endophytes germinate as thin-walled hyphae, suggesting to Duckett et al. [77] that they are perennating structures functionally equivalent to vesicles produced by Glomeromycota. Long ignored, Glomerales intraradical vesicles clearly function as propagules [84], explaining why these fungi can survive as endophytes in living roots for up to 10 years after arbuscules collapse [81]. Thus homology between lipoid-mycosomes and the lipoid vesicles of Mucoromycotina and Glomeromycota may be a reasonable expectation.

In conclusion, we describe mycosome life history states derived from plants and diverse endophytic fungi, show that these unique propagules revert to walled cells, and can be cultured to further reveal their morphology, genetic structure and biological roles within plants, fungi and animals. Future tests of mycosome concepts, particularly PMO origin and function, will benefit from precise methodologies that regulate in vitro mycosome formation and reversion to the walled state. With these skills in hand, endophytic fungi should reveal the details of a highly regulated, intracellular life history phase that may significantly alter our views of plant and fungal evolution.

## Supporting Information

File S1 Includes Table S1, media components, Table S2, experimental conditions for text figures, and Figures S1 to S5.
(DOCX)
