# Supplementary material for: Novel Symbiotic Protoplasts Formed by Endophytic Fungi Explain Their Hidden Existence, Lifestyle Switching, and Diversity within the Plant Kingdom
Source: PLoS One. 2014 Apr 28;9(4):e95266. doi: 10.1371/journal.pone.0095266 (PMC4002429; doi:10.1371/journal.pone.0095266)
Supplement: File S1 — Includes Table S1, media components, Table S2, experimental conditions for text figures, and Figures S1 to S5. (DOCX) [file pone.0095266.s001.docx]

Supporting Information File S1: Tables S1, S2 and Figures S1 to S5

Atsatt & Whiteside, 4-5-2014

Table S1: Media Components

| CAN | A non-buffered acidic medium (pH @3.6) containing 50 mg citrate and 100 mg ammonium nitrate in one liter of deionized water, autoclaved, to which erythromycin (0.1mg/ml) was added prior to use. |
| --- | --- |
| MsM | 0.1g NH_4_NO_3_, 0.5g KH_2_PO_4_, 0.3g CaCl_2_-2H_2_O, 0.025g NaCl, 0.020g FeEDTA, 30g MgSO_4_-7H_2_O, 20g mannitol, 10 ml glycerol, in one liter deionized water. Autoclaved. |
| MsM + Soy | Bacto-Tryptic Soy Broth without Dextrose: 27.5 g/L deionized water, autoclaved. A 10 or 20 percent volume was added to autoclaved MsM. |
| 2xT864 | 2xT864 contains Murashige & Skoog high nitrogen basal salt mixture, plus casein enzymatic hydrolase, glycine, *myo*-inositol, kinetin and vitamins. A one-liter packet was dissolved in 500 ml water and autoclaved. IAA was added in some experiments. (phytotechlab.com) |
| Yolk-HCM  (High  Calcium-  Magnesium) | 15% nitrogen (13.7% nitrate nitrogen, 1.3% ammonium nitrate), zero phosphorus, 15% soluble potash, 9.1% water-soluble calcium, 1% magnesium, 0.015% boron, 0.015% copper, 0.075% iron, 0.03% manganese, 0.015% molybdemum and 0.015% zinc (Sun Gro Horticulture, Technigro 15-0-15 water soluble fertilizer). Adjusted to 200 ppm N (1.33g/l) and autoclaved. |

Table S2: Experimental conditions for Figures 1-10

| **Figure 1.** | (**a)** *Psilotum* cytoplasm allowed to leak from three surface sterilized freshly cut stem segments floated in wells containing 5 ml AC medium + BSA: stems removed at 48 hr. One ml of filter-sterilized prune extract added to each well **(**Atsatt, 2003), observed at 7 days. **(b-c)** *A. pullulans* hyphae cultured 14 months in 300 ml deionized water plus erythromycin. |
| --- | --- |
| **Figure 2.** | **(a** and **c)** from Atsatt (2003) with permission. **(b-e)** *A .pullulans* yeast cells cultured from post-reproductive *Psilotum* stems dried 24 hr on bench top, surface sterilized, cell extract 24 hr in water. **(f-g)** *A. pullulans* yeast cultured from surface sterilized *Cuscuta subinclusa* stems 5 days moist at 6^o^ C, cell extract 48 hr in water over YM agar. **(h)** *A. pullulans* yeast cultured from post-reproductive surface sterilized *Psilotum* stem segments floated 48 hr in water, cell extract cultured 40 hr in BMGYM (see Atsatt 2003) over YM agar. |
| **Figure 3.** | (**a,** **c** and **d**) *A. pullulans* yeast cultured from *Cuscuta subinclusa* haustorial coils, stem coils surface sterilized, pretreated 40 hr in CAN, macerated in fresh CAN, cell extract cultured 72 hr prior to fixation. (**b**) *Psilotum* stems surface sterilized, 48 hr in sterile water, cell extract 45 hr in BMGYM.  (**e** and **f**), *Psilotum* stems surface sterilized, 73 hr in sterile water, cell extract 46 hr in BMGYM. |
| **Figure. 4.** | Right Panel. **(a)** *Cuscuta* stem cell extract 24 hr in MsM. **(b)** *Rhodotorula* cells 2 months in CAN, filtered 0.8 µm into MsM + 2 mg/L IAA for 20 hr. **(c and e)** Glycerol-frozen *Saccharomyces* cells 48 hr on YPD agar, 24 hr in MsM. **(d)** *Aureobasidium* 6 days in MsM +10% Soy, Ms filtered into same medium for 7 days. **(f)** An unpublished photo from Atsatt, 2003. **(g)** *Filobasidium* cells 3 months in CAN, mycosomes filtered 0.8 µm into *Psilotum* cell extract + 10 ml CAN; 24 hr at 6^0^ C + 24 hr room temp. *Psilotum* extract controls were negative for fungi. |
| **Figure 5.** | **(a-b)** *Cr. victoriae* cells 7 days in CAN. **(c)** *Cr. stepposus* cells 12 days in CAN. **(d)** *Cr. stepposus* cells 40 days in CAN, transferred to 2xT864 for 20 hr. **(e)** *Taphrina* cells 6 days in MsM + 2 mg/L IAA. **(f)** *Rhodotorula* cells 14 days in CAN + 1mg/L IAA. **(g-i)** *Penicillium* conidia 48 hr in CAN. **(j)** *Cladosporium* conidia 24 hr in CAN. **(k)** *Penicillium* conidia 48 hr in 2xT864. **(l)** *Penicillium* conidia 72 hr in 2xT864 + 2 mg/L IAA, (Ms sacs from young hyphae tips). **(m)** *Taphrina* cells 7 days in CAN, mycosomes filtered into half strength M524 medium over half strength YM agar. **(n)** *Aureobasidium* yeast 5 days in MsM+20% Soy, transferred to MsM for 14 days. **(o-p)** *Rhodotorula* cells 21 days in MsM. **(q-r)** Glycerol-frozen *Saccharomyces* cells 48 hr on YPD agar, 48 hr in 50%CAN-25%MsM-25%Soy. **(s)** *Taphrina* cells 21 days in MsM. **(t-v)** *Taphrina* cells 21 days in MsM without glycerol. |
| **Figure 6.** | **(a)** *Rhodotorula* cells 8 days in CAN + 2 mg/L IAA and 2 mg/L kinetin; mycosomes filtered into 0.1g BSA in 100 ml CAN over YM agar, 7 days dark. **(b)** *Rhodotorula* mycosomes from (a) filtered into 2 g/L BSA + 3 mg/L IAA + 3 mg/L kinetin for 48 hr. **(c)** *Rhodotorula* cells 21 days in CAN + 1 mg/L IAA: 3 ml filtered into BMGYM (see Atsatt 2003) plus 2 mg/L kinetin, 1 mg/L IAA, 2 mg/L glycine, over YMA, observed at 24 hr. **(d)** Ms-containing medium from **(e)** flooded over YM agar for 24 hr. **(e)** *Mycosphaerella* conidia 5 days in 75% MsM-25% Soy. |
| **Figure 7.** | **(a-b)** *Taphrina* cells 48 hr in CAN, Ms filtered 0.8 into M524 basal salts for 48 hr. **(c-d)** and **(g-i)** *Rhodotorula* cells 2 months in CAN, Ms filtered into modified MsM (without MgSo_4_, mannitol and glycerol) plus 2 mg/L IAA) for 24 hr. **(e-f)** *Rhodotorula* cells cultured 30 days in 2xT864 + 2 mg/L IAA. **(j-l)** California kiwifruit cell extract cultured 6 days in MsM. |
| **Figure 8.** | All stems surface sterilized. (**a-e)** Extract from fresh (no cold treatment) young *Psilotum* stem tips, macerated for 22hr (**a** and **c**) and 45 hr (**b**, **d**, **e**) in MsM. **(f-h)** Young *Psilotum* stems sealed moist 10 days at 6^O^ C, macerated in MsM + 20% Soy, sampled at 2 hr (**f**) and 24 hr (**g** and **h**). **(i -j)** Young *Psilotum* stems 7 days moist at 6^0^ C, stems floated 24 hr in MsM, macerated in MsM 24 hr. **(k-n)** Post-reproductive *Psilotum* stems sealed moist for 7 days at 6^o^C, macerated in MsM, sampled at 24 (**k**) and 72 hr (**l**-**n**). |
| **Figure 9.** | **(a)** *Taphrina* mycosomes filtered 0.8 µm into M541 + 40%YM broth. **(b)** *Penicillium* mycosomes filtered 0.8 µm into 2xT864 medium. **(c)** *Fusarium* conidia 21 days in CAN, Ms filtered into MsM medium + 2 mg/L kinetin. **(d)** *Rhodotorula* cells 4 weeks in 2xT864 + 2 mg/L IAA. **(e-f)** *Rhodotorula* cells 14 days in CAN + 1mg/L IAA, mycosomes filtered into 2 g/L BSA (bovine serum albumin) + 1 mg/L IAA for 48 h. **(g)** *Taphrina* cells cultured 26 days in 2xT864 + 2 mg/L IAA. **(h)** *Filobasidium* ms filtered 0.8 µm into *Psilotum* cell extract for 24 hr, chloroplast-cytoplasm settled, liquid above replaced with 10^-6^ M ethylene for 48 hr. **(i)** *Rhodotorula* cells cultured 30 days in 2xT864 + 2 mg/L IAA.  **(j and n)** Kiwifruit cells and cell extract cultured 7 days **(j)** and 72 hr **(n)** in MsM. **(k)** *Trichoderma* from Day Lily mycosomes co-cultured with *Staphylococcus warneri*. **(l)** *Taphrina* mycosomes filtered 0.8 µm into *Psilotum* cell extract. **(m)** *Penicillium* from apple cell extract cultured in CAN. **(o-q)** *Cladosporium sp.* from 2xM541 supplemented with 2 mg/L IAA. The packaged medium contained contaminant fungal spores, and was not autoclaved. |
| **Figure 10.** | **(a-b)** 5 ml mycosomes from 100 ppm N added to 20 ml water containing 10^-6^ M ethylene + 1 ml YM broth for 24 hr. **(c)** 1 ml yolk-water in 20 ml water containing 100 ppm N +10-8 M ethylene + 2 mg/L IAA. **(d-e)** Yolk-water 48 hr cold, 1 ml added to 20 ml MsM for 7 days, sample then flooded over Sabouraud dextrose agar for 4 days. **(f-k)** This treatment was unusual and produced reticulate protoplasts with clear reversion to *R. glutinis*. It resulted from a low mycosome inoculum level: after 24 hr in 100 ppm N medium, a small (200µl) sample was transferred to 20 ml water for 38 days. |

**
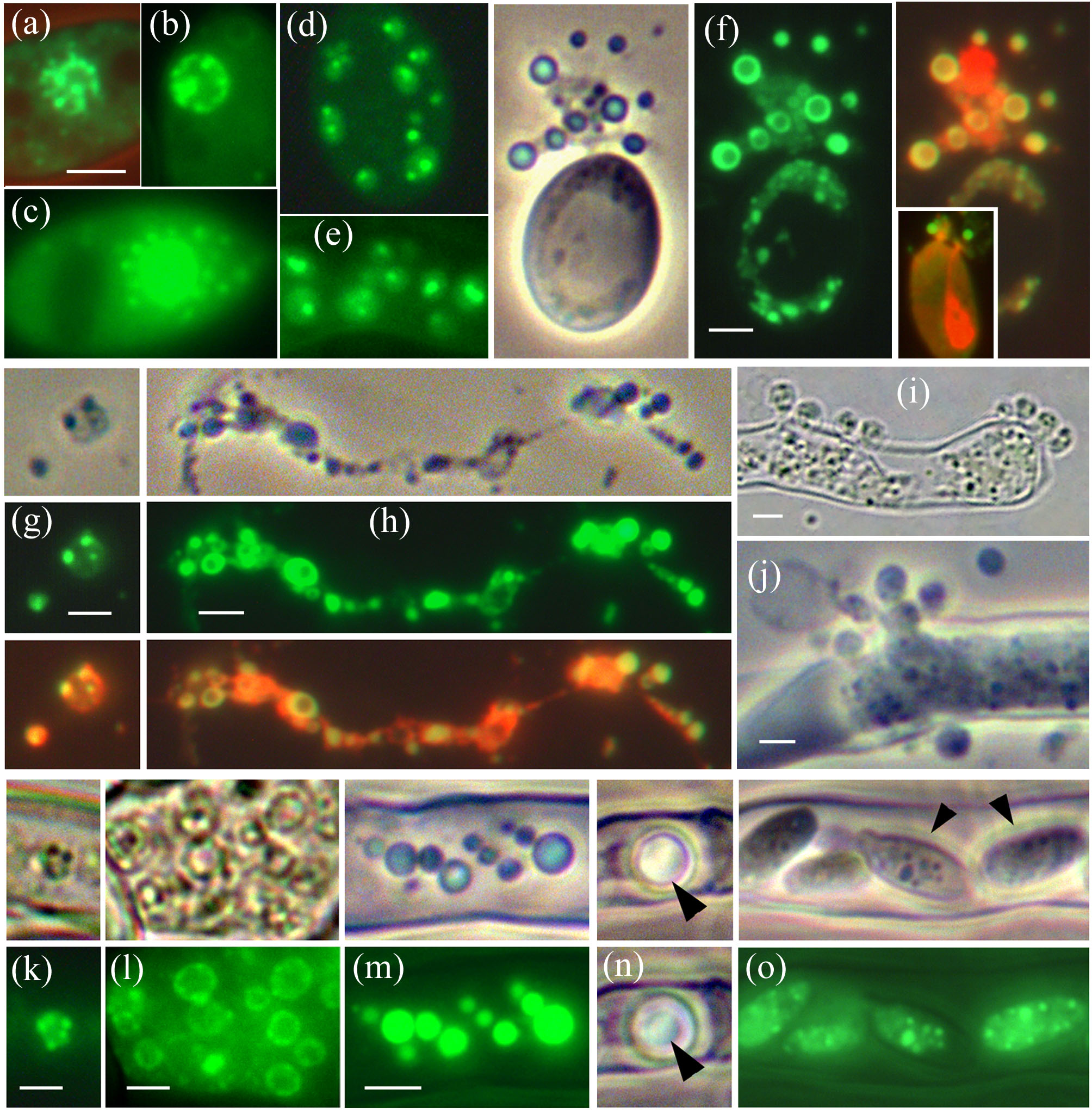
**

**Figure S1. Mycosomes observed within *Aureobasidium* cells**. (a-e) Early Ms development within PMOs may be observable with SYTO 9 staining: (a, b) condensed-Ms may originate within an invaginating spheroid organelle, or (c) bud from the organelle margin. (d-e) The Ms-plasmalemma apparently expands as a cytoplasmic sac, within which Ms divide: (d) a yeast cell, (e) a hypha. (f-h) Phase contrast, green and red fluorescence channels: (f, left) Ms-containing membrane sacs; (f, center) nucleic acid staining within the protoplast; (f, right) red PI entry into the membrane sacs: (inset) sac remained inside, Ms release via an exit tube. (g-h) Type I protoplasts (g) and filaments (h) observed in culture media. (i-j) *A. pullulans* hyphae releasing Ms-protoplasts through wall openings (i, bright field; j, phase contrast). (k-n) Type II Ms observed in hyphae, phase contrast and SYTO 9 fluorescence. (k-l) Vacuolate-Ms express punctate bodies within the narrow protoplast. (m) Budding lipoid-Ms. (n) A lipoid-Ms at two focal depths; the PMO lipoid body (top arrowhead) conceals an internal body (bottom arrowhead). (o) Walled endospores develop within the vacuolate PMO of elongated mycosomes (arrowheads). Bars = 2.0 µm. (a-e) use bar in (a).

**Experimental: (a-e)**, *Aureobasidium* yeast and hyphae 4-7 days in 80% CAN-10% MsM-10% Soy. **(f-h)** *Aureobasidium* yeast 5 days in 80% MsM-20% Soy; transferred to MsM for 14 days. **(i-o)** First generation *Aureobasidium* hyphae from surface sterilized *Psilotum* stems floated in 80% MsM-20% Soy at 6^o^C for 9 days: **(i-m)** Stem extract 3 days, and **(o)** 4 days in 80% MsM-20% Soy. **(n)** *Aureobasidium* hyphae 48 hr in water over Sabouraud agar.

**Figure S1 Discussion.** Differentiation of PMOs (Fig. S1a-c), and further mycosome development may be stimulated by culture in acidic media (CAN) combined with low percentages of MsM and Soy. We have not attempted to repeat or refine this result. As revealed by SYTO 9 staining, both yeast and hyphae expressed spheroid organelles that contained green-fluorescing punctate bodies, thought to be condensed-Ms formed by membrane invagination (a-b), or by budding from the organelle margin (c). Neighboring yeast cells (d) and hyphae (e) contained only the small punctate bodies dividing within a fluorescing membrane sac, presumably the plasmalemma of Type I mycosomes (see g). At later stages, one or more mycosome-containing sacs were released through cell wall openings with normal cover slip pressure (f). Within the culture medium, mycosomes developed into Type I sacs (g) and mycosome-forming filaments (h). PI entry into the protoplast-plasmalemma may result from membrane damage (slide preparation) and/or because this membrane is unusually thin. Protoplasts emerging from *Aureobasidium* hyphae (i-j) also appear to be formed endogenously, rather than as buds cut off from the parent protoplast.

Cultured hyphae show two mycosome types: vacuolate-Ms that contain fluorescing punctate bodies within the narrow protoplast (k, l), or more commonly, budding lipoid-Ms (m) that fluoresce solid green, perhaps because they contain an internal body as in (n). Viewed at two focal depths, this large Type II Ms shows a PMO containing a refractive lipoid-body (top arrowhead), which conceals an internal body (bottom arrowhead). As endospores develop (o), lipids are no longer present and the walled cells occur in a clear space (presumably the PMO vacuole) within the Type II mycosomes (arrowheads).


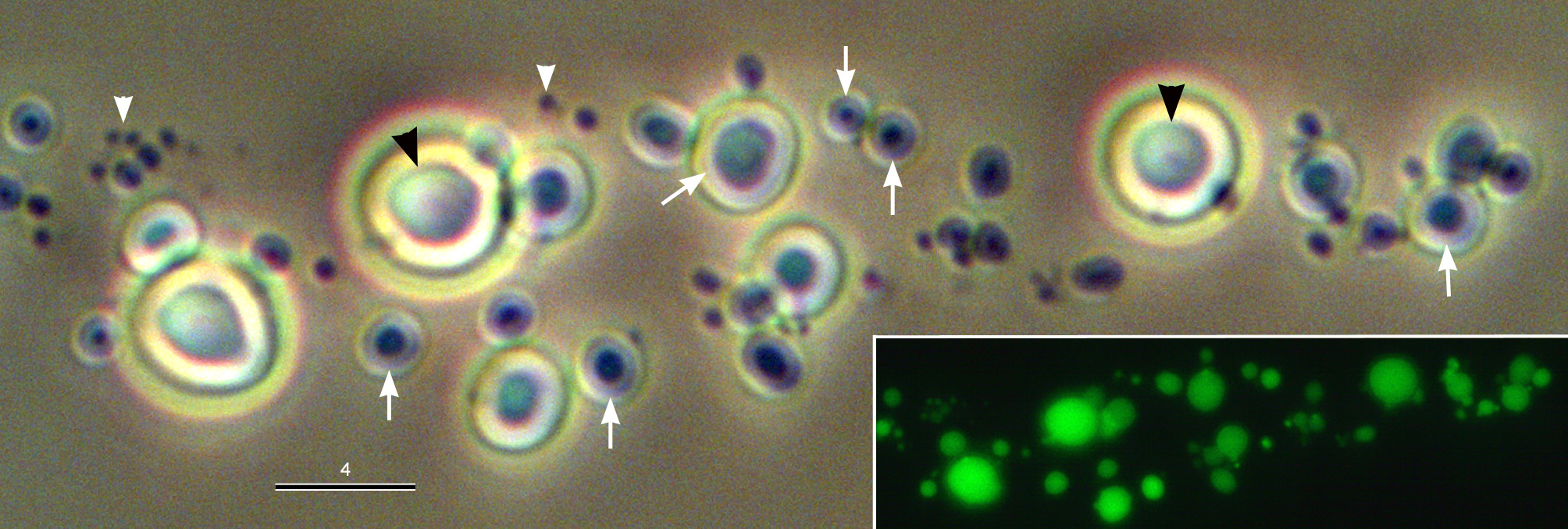


**Figure S2: Mycosomes cultured from the lichen fungus *Ramalina conduplicans*.** Condensed-Ms (white arrowheads) differentiate as intermediate stages that express a dark punctate body that enlarges within a lipoid-body (white arrows). At later stages the depleted lipoid-body (black arrowheads) remains visible around the internal body, which potentially may differentiate as a walled cell within the vacuolate PMO. Note Ms budding from the ring-like protoplast. Stained with SYTO 9 (inset), the mycosomes are positive for nucleic acids. Bar = 4.0 µm.

**Experimental:** The *R. conduplicans* culture was provided by Jae-Seoun Hur; Korea Lichen Research Institute. Fungal hyphae were received in a liquid growth medium and transferred to MsM for 24 h.

**
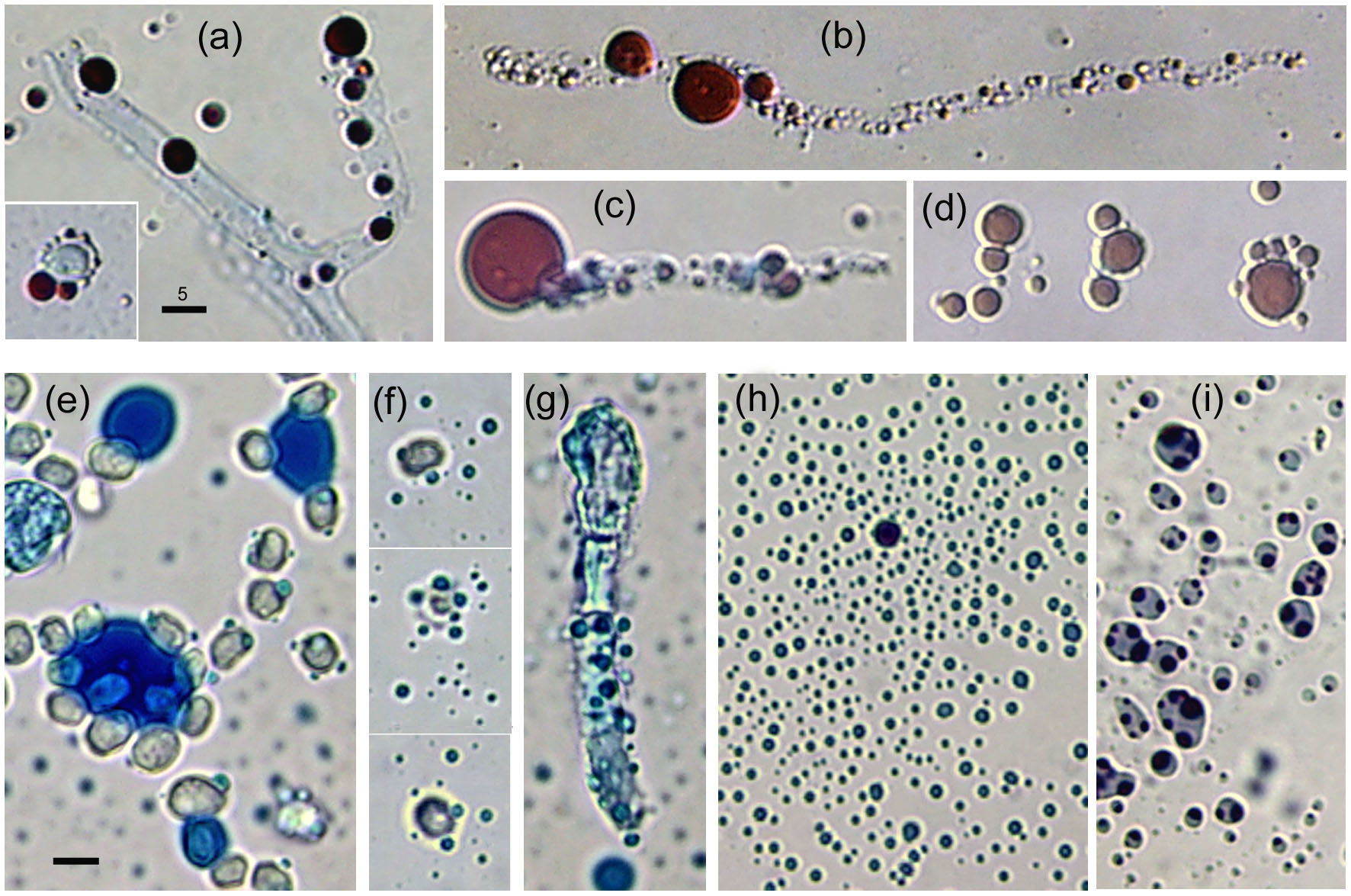
**

**Figure S3**. **Mycosomes from *Mucor* and *Mortierella* spores and hyphae**.

**(a-d) *Mucor* sp; AB/SIV stain**. (a) Lipoid-Ms were released from cultured hyphae and spore cell walls (inset). (b) An acytoplasmic filament containing numerous mycosomes and enlarging Type II lipoid-Ms. (c) A ‘germinating’ Type II lipoid-Ms forming a protoplast-filament. (d) Typical budding from the margin of Type II-Ms. **(e-i) *Mortierella* sp.** (e) Non-staining *Mortierella* spores were associated with AB-staining Type II-Ms. (e-g) AB-staining mycosomes were released from spore margins (e-f) and hyphae (g). (h-i) Filtered to remove parent cells, mycosomes from (e-g) responded differently to two agar types, reproducing large numbers of condensed mycosomes when flooded over ½ strength YM agar (h), and differentiating as protoplasts that contain lipoid-bodies (i), in response to agar containing ½ strength Soy medium. Bars in (a) and (e) = 5.0 µm in all photos.

**Experimental:** (a) 2xT864 medium + 2 mg/L IAA, autoclaved, medium inoculated with *Mucor* spores and hyphae, observed at 28 days; (b-c) 2x541 medium + 2 mg/L IAA, autoclaved, medium inoculated with *Mucor* spores and hyphae, observed at 30 days; (d) Medium sample from (a) above, transferred after 6 days to phosphate buffered saline over YM agar for 24 hr. (e-g) *Mortierella* spores and hyphae transferred from YM agar to Soy-MsM for 48 hr, mycelium-mat removed and liquid sampled; (h) Soy-MsM from (e-g) filtered 0.8 µm into fresh Soy-MsM, half of the filtrate flooded over ½ strength YM agar for 24 hr; (i) the remaining half flooded over agar containing ½ strength Soy medium.


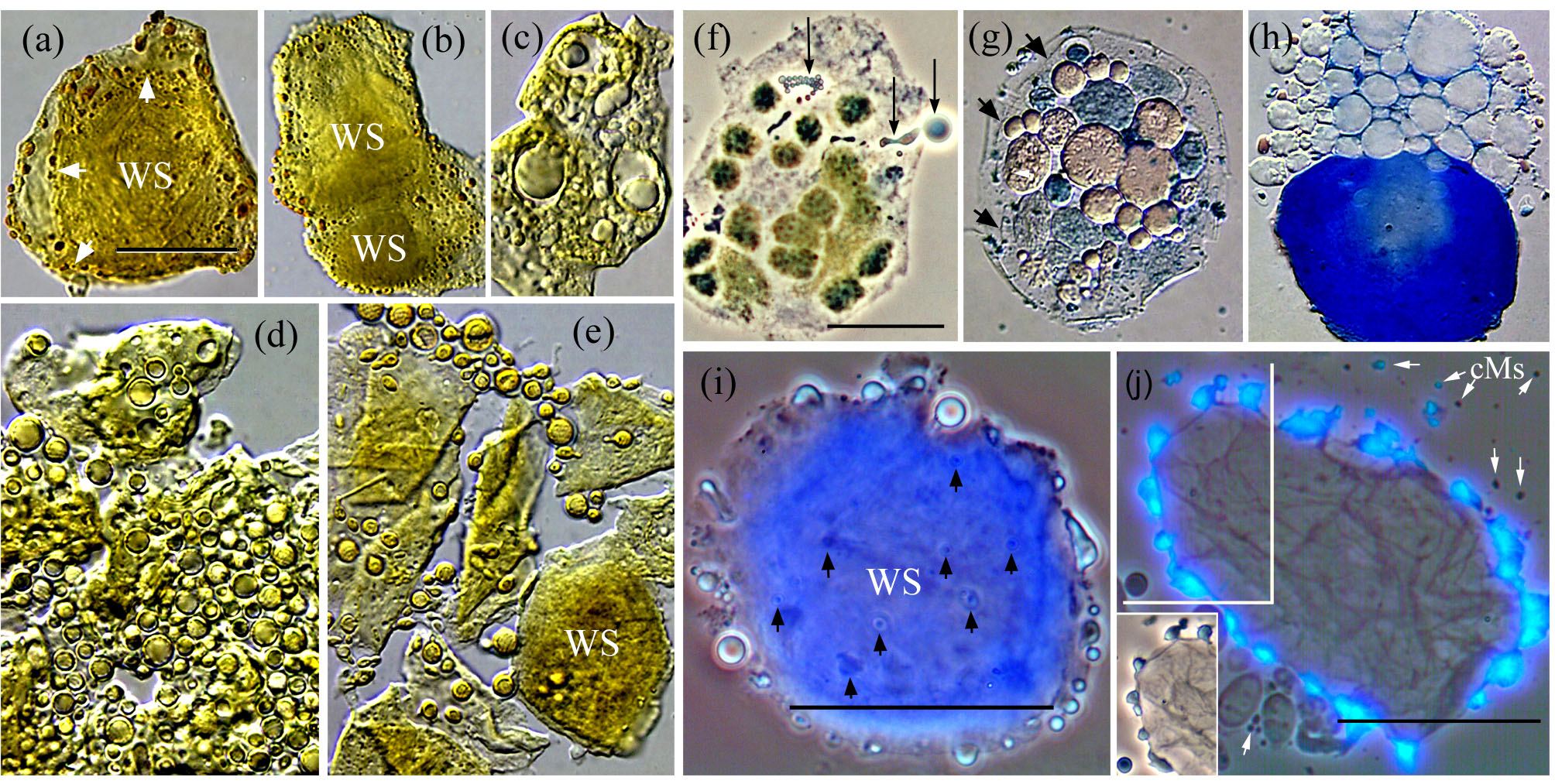


**Figure S4. Mycosomes enlarge as giant protoplasts that form an internal walled-sporangium.**

(a-e), Walled sporangia (WS) from *Filobasidium* Ms filtered 0.8 µm, MR stain. (a) Ms are present at the protoplast margin; white arrows indicate the margin of the internal WS. (b) The protoplast contains two WS and numerous peripheral Ms. (c) A wall-less sporangium, showing lipoid-bodies within vacuole-like compartments. (d) Numerous Type II Ms developing within a cluster of sporangia. (e) Budding Ms and yeast cells associated a fragmented cluster of sporangia. (f) A similar protoplast type from *Psilotum nudum* cell extract that contains numerous chloroplasts. Note Ms (arrows) that apparently derive from the chloroplasts. (g-h) *Rhodotorula* protoplasts stained with AB/SIV. (g) The inner sporangium membrane (arrows) contains aberrantly developing lipoid and cytoplasmic Ms. (h) Ms-starch grains pressed out of a WS. The central cluster of starch grains is contained within an AB-staining protoplast. (i-j) Protoplasts photographed with combined fluorescence and phase contrast channels: (i) The internal cell wall fluoresces blue with Fungi Fluor stain. Internal mycosomes are visible (arrows) and refractive lipoid-Ms are associated with the outer membrane. (j) A large *Taphrina* protoplast, surrounded by fluorescing DAPI (4', 6-diamidino-2-phenylindole)-stained lipoid-Ms (inset shows outlined section in phase contrast photo). Note that parent yeast cells (bottom) and some condensed-Ms (cMs, white arrows) do not show fluorescing nuclei. All bars = 20 µm. a-h, use bar in a or f**.**

**Experimental:** (a-e) *Filobasidium* cells 2 months in CAN, Ms filtered into *Psilotum* cell extract in 10 ml water + 10 ml CAN, 24 hr at 6^0^ C + 24 hr room temp. (f) Sterilized *Psilotum* stems 48 hr in water, cell extract 14 days in 100 ml water containing 0.3g BSA, 2 ml glycerol and 10^-^6 M ethylene. (g) *Rhodotorula* cells 5 days in CAN + 2 mg/L kinetin + 1 mg/L IAA, filtered into water over YM agar. (h) *Rhodotorula* cells 20 days in CAN plus 0.5g/L glucose. (i) 5 ml egg yoke mycosomes in 100 ppm N fertilizer added to 10 ml water containing 10^-5^ M ethylene for 7 days. (j) *Taphrina* cells cultured 9 days in MsM.

**
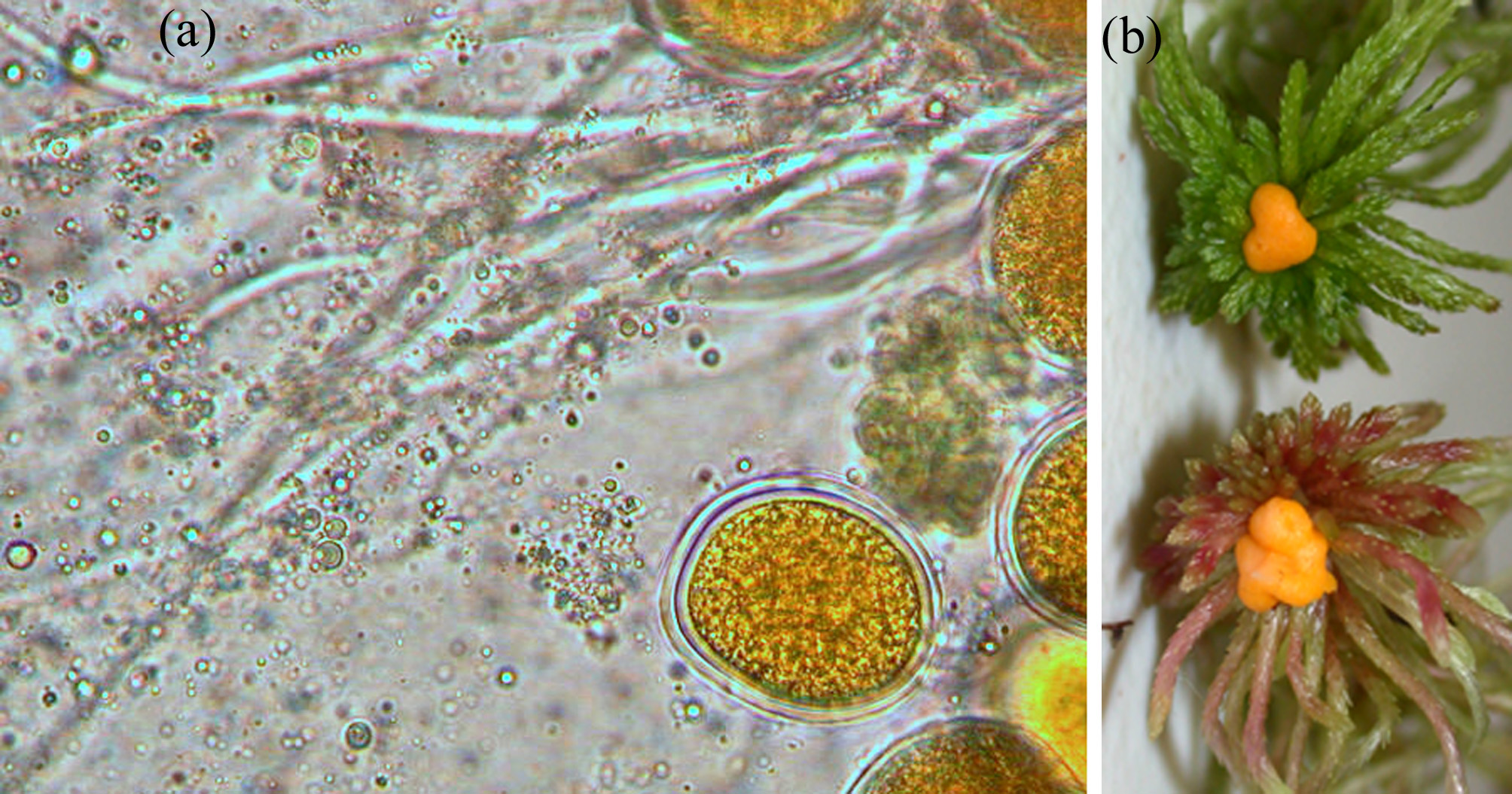
**

**Figure S5: Putative mycosomes (a) from *Endogone pisiformis* (Mucoromycotina), sporulating on healthy *Sphagnum* moss (b).** (a) The enlarged photo shows double-walled yellow sporangia (right) and putative mycosomes associated with narrow thin-walled vesicular hyphae (left). The background is color-adjusted to emphasize the lipoid-Ms.

Collected and photographed by Tom Volk, Howe Demonstration Forest, Gander Lake, Newfoundland, Canada. *From (http://en.wikipedia.org/wiki/User:Sasata/Endogone).*
